# Spatio-Temporal Expression Patterns of *Arabidopsis thaliana* and *Medicago truncatula* Defensin-Like Genes

**DOI:** 10.1371/journal.pone.0058992

**Published:** 2013-03-18

**Authors:** Mesfin Tesfaye, Kevin AT. Silverstein, Sumitha Nallu, Lin Wang, Christopher J. Botanga, S. Karen Gomez, Liliana M. Costa, Maria J. Harrison, Deborah A. Samac, Jane Glazebrook, Fumiaki Katagiri, Jose F. Gutierrez-Marcos, Kathryn A. VandenBosch

**Affiliations:** 1 Department of Plant Biology, University of Minnesota, Saint Paul, Minnesota, United States of America; 2 Department of Biological Sciences, Chicago State University, Chicago, Illinois, United States of America; 3 Boyce Thompson Institute for Plant Research, Ithaca, New York, United States of America; 4 Department of Plant Sciences, University of Oxford, Oxford, United Kingdom; 5 School of Life Sciences, University of Warwick, Coventry, United Kingdom; 6 US Department of Agriculture-Agricultural Research Service-Plant Science Research Unit, Saint Paul, Minnesota, United States of America; University of Minnesota, United States of America

## Abstract

Plant genomes contain several hundred defensin-like (*DEFL*) genes that encode short cysteine-rich proteins resembling defensins, which are well known antimicrobial polypeptides. Little is known about the expression patterns or functions of many *DEFL*s because most were discovered recently and hence are not well represented on standard microarrays. We designed a custom Affymetrix chip consisting of probe sets for 317 and 684 *DEFL*s from *Arabidopsis thaliana* and *Medicago truncatula,* respectively for cataloging *DEFL* expression in a variety of plant organs at different developmental stages and during symbiotic and pathogenic associations. The microarray analysis provided evidence for the transcription of 71% and 90% of the *DEFL*s identified in Arabidopsis and Medicago, respectively, including many of the recently annotated *DEFL* genes that previously lacked expression information. Both model plants contain a subset of *DEFL*s specifically expressed in seeds or fruits. A few *DEFL*s, including some plant defensins, were significantly up-regulated in Arabidopsis leaves inoculated with *Alternaria brassicicola* or *Pseudomonas syringae* pathogens. Among these, some were dependent on jasmonic acid signaling or were associated with specific types of immune responses. There were notable differences in *DEFL* gene expression patterns between Arabidopsis and Medicago, as the majority of Arabidopsis *DEFL*s were expressed in inflorescences, while only a few exhibited root-enhanced expression. By contrast, Medicago *DEFL*s were most prominently expressed in nitrogen-fixing root nodules. Thus, our data document salient differences in *DEFL* temporal and spatial expression between Arabidopsis and Medicago, suggesting distinct signaling routes and distinct roles for these proteins in the two plant species.

## Introduction

Defensins are a large family of endogenous antimicrobial polypeptides present in most eukaryotic life forms [1], [2]. These small, charged, cysteine-rich polypeptides are secreted or sequestered within compartments of the endomembrane system and have broad-spectrum anti-fungal, anti-bacterial, anti-viral, and/or insecticidal activity via a variety of proposed molecular mechanisms [1], [3], [4], [5], [6].

Plants have a large repertoire of genes encoding defensin-like (DEFL) polypeptides that have a conserved pattern of cysteine residues, but are otherwise highly variable in the mature protein [7], [8], [9]. The model legume *Medicago truncatula* (hereafter called Medicago) has at least 778 *DEFL* genes [7], [8], [10], [11], [12]. Arabidopsis has 317 *DEFL* genes [9] while rice has 93 *DEFL* genes [8].

*DEFL* gene families appear to have expanded and diversified in plant genomes via tandem and ectopic duplication followed by successive rounds of diversifying selection [7], [9], [13]. Based on sequence diversity, the *DEFL*s have been classified into more than 100 different subgroups [8]. The expansion and diversification of the *DEFL* family appear to have resulted in some divergent subgroups of *DEFL*s characteristic of different plant lineages, while other subgroups such as the defensins themselves have remained broadly conserved among lineages [8].

The phylogenetic diversity among *DEFL*s suggests that different gene family members may play distinct roles in divergent taxa, but knowledge of the functions of this large gene family is largely unknown. In contrast, a number of roles for plant defensins have been identified. Some are constitutively expressed or developmentally regulated and act as antimicrobial agents that protect nutrient-rich and vulnerable tissues in seed development and seedling growth [3], [14]. Other defensins are deployed specifically during defense responses and are induced downstream of jasmonate or other defense signaling molecules [15], [16]. Roles for *DEFL*s in plant-microbe interactions are likely to be varied. For example, nodule cysteine-rich proteins (NCRs) belonging to the *DEFL* family were recently demonstrated to effect the terminal differentiation of nitrogen-fixing bacteroids in root nodules of certain legumes [17].

Some members of the highly diverse *DEFL* group have been shown to function in plant development [18]. For example, some S-locus cysteine-rich proteins (*SCR*s), which make up a subgroup of *DEFL*s, are expressed in pollen tubes and act as signal molecules in self-incompatibility in some dicots [19], [20]. Other *DEFL*s have been identified that mediate pollen tube development and guidance [21], [22]. Published evidence suggests that *DEFL*s act in a variety of plant development processes [6], [23], [24], [25], [26]. However, classifying *DEFL*s as functioning either in microbial interactions or in plant developmental regulation may be misleading. A tomato defensin shows evidence of bifunctionality, acting both to regulate plant development and to confer disease resistance [27].

Despite great progress towards creating gene expression atlases for Arabidopsis [28] and Medicago [29], gene expression data for *DEFL*s are far from comprehensive in either species. This is because standard Affymetrix arrays utilized to construct gene atlas projects contain only 12% (Arabidopsis ATH1 array) and 50% (Medicago genome array) of known *DEFL*s (Silverstein, unpublished). Although deep transcriptome sequencing via RNA-seq methodologies have become routine for expression profiling, the highly redundant nature of the DEFL gene family poses serious problems for mapping short sequence reads. Hence, it has been difficult to ascertain comprehensive expression patterns of *DEFL*s as a family and to determine whether *DEFL* functions are conserved or divergent among angiosperms.

To address this gap, we designed a custom Affymetrix microarray (here after called the AtMtDEFL array) with probe sets for known and predicted Arabidopsis and Medicago *DEFL*s and the closely-related family of maternally expressed genes (*MEG*s) [8], [30]. The custom array was utilized for hybridizations of mRNAs from a wide range of conditions and tissues from both plant species, enabling a comparative cataloging of *DEFL* gene expression in the two model plant species. The custom chip analysis of *DEFL* transcript abundance provided the first expression evidence for many of the computationally identified *DEFL*s previously lacking gene expression evidence, defined both common and unique patterns of *DEFL* gene expression between Arabidopsis and Medicago, and identified organ-specific and microbe-induced family members.

## Results

### Development of the Custom AtMtDEFL Array and Robust Data Normalization Methods

The AtMtDEFL array includes probe sets for 317 Arabidopsis *DEFL*s, 15 Arabidopsis *DEFL*-related Genes (*MEG*s) [8], [30], and 684 Medicago *DEFL*s [8], plus additional marker genes and probe sets with invariant levels of expression (hereafter called invariant genes) to aid microarray data normalization (See Materials and Methods for details). Probe sets were interspersed on the custom array although chip hybridization and microarray data analysis were performed for only one plant species at a time. The subset of the AtMtDEFL array made up of Arabidopsis probe sets is hereafter referred to as the AtDEFL array whereas the subset of the AtMtDEFL array made up of Medicago probe sets is hereafter termed the MtDEFL array.

**Figure 1:**
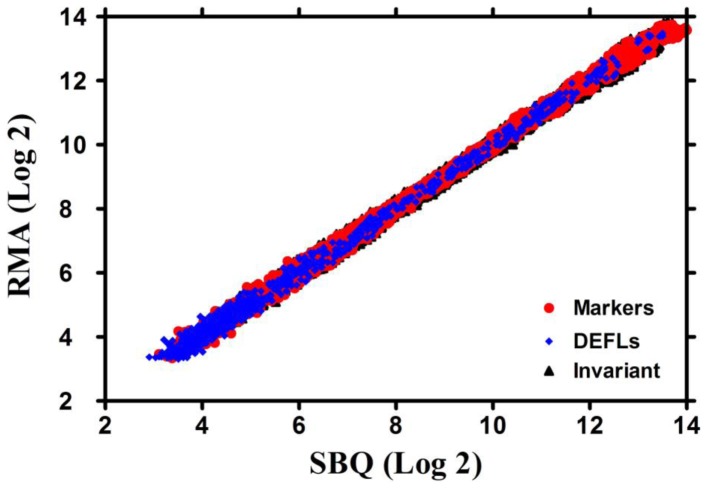
Scatter plots of SBQ normalized gene expression values compared to whole-array RMA across 36 ATH1 arrays. Only a subset of 299 probe sets that correspond to genes represented on the AtMtDEFL array (37 DEFLs, 171 invariants, 91 marker genes) were used for SBQ normalizations. Each was compared to the reference RMA normalization, which included all 22,810 probe sets on the Affymetrix ATH1 array in the normalization process. Expression values were log_2_ transformed. All 37 probe sets on the ATH1 array that matched an Arabidopsis *DEFL* with at least 6 of 11 exact-match probes per probe set were included in the analysis. GEO accessions for ATH1 arrays included three biological replicates of: GSE1491 (seedlings), GSE5630 (cotyledons, leaves, senescent leaves), GSE5631 (roots), GSE5632 (carpels, stage 9 flowers, stamens), GSE5633 (stems), GSE5634 (old and young siliques), and GSE7227 (seeds).

**Figure 2:**
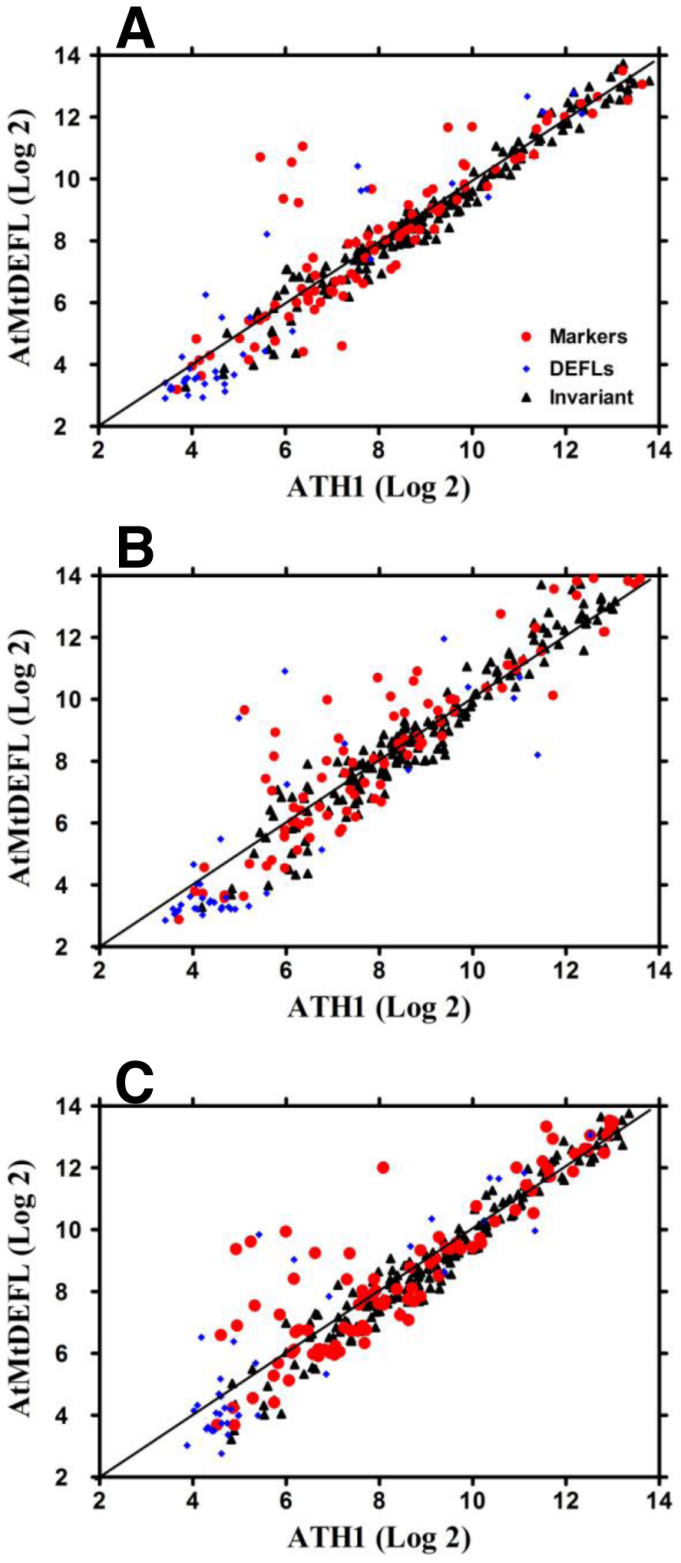
Scatter plots showing correlation of SBQ normalized AtMtDEFL array expression values compared to publicly available GEO ATH1 array data. A, roots. B, leaves. C, inflorescences. Probe sets for 171 invariants and 91 marker genes are identical in AtMtDEFL and ATH1 arrays. The 37 *DEFL* probe sets were represented with different probes on the two arrays with differences in some cases in the underlying gene model. Log_2_ signals for biological replicates were averaged prior to cross-platform comparison. GEO accessions for ATH1 arrays included: GSM131558, GSM131559, GSM131560 (roots); GSM131498, GSM131499, GSM131500 (leaves); and GSM62694, GSM227612 (inflorescences).

Microarray data normalization algorithms such as Affymetrix's MAS 5.0 [31] or loess [32] require that the majority of genes show an unchanged pattern of expression among the conditions under consideration or at least that an equivalent proportion of genes are up- and down-regulated. Alternatively, RMA and other quantile normalization based schemes [33], [34] require that the density distribution of intensities is at least qualitatively similar across samples. These essential probe intensity distribution assumptions for microarray data are clearly violated when analyzing data from boutique arrays such as the AtMtDEFL chip used in this study. For example, we observed more than 500 Medicago *DEFL*s expressed in nitrogen-fixing root nodules, yet only a minority was detectable in uninoculated roots. These extreme expression patterns pose a challenge to routine microarray data normalization algorithms [35], [36]. In this study, we evaluated three microarray normalization algorithms: the Stable-Based Quantile (SBQ; [36]), RMA with Invariant Median Scaling (RIMS), and RMA with Median Absent Probe set Scaling (RMAPS) (See Materials and Methods, Text S1, and Figures S5, S6, S7, S8, S9, S10, S11 for details). The Stable-Based Quantile (SBQ) approach gave the best performance for the custom chip and despite being restricted to the very small set of genes on the AtMtDEFL array exhibited very tight correlation with full-scale RMA normalization data that utilizes a genome-wide set of genes (Figures 1 and 2, Figure S1). Consequently, we used SBQ to normalize AtMtDEFL custom array datasets in the subsequent studies.

### Validation of Arabidopsis Microarray Data

We employed quantitative reverse-transcription polymerase chain reaction (qRT-PCR) to verify the AtDEFL microarray data. Using geNorm ranking [37], of expression stability of candidate reference genes (details in Materials and Methods) we initially identified four candidates as potential reference genes (Figure S2A). Gene expression stability analysis using qRT-PCR assays showed all four genes to be suitable for transcript quantification (Figure S2B). We selected two of the Arabidopsis genes, *metallothionein 2B* (At5g02380) and *yellow-leaf-specific gene 8* (At5g08290), as reference genes for qRT-PCR validation of microarray data.

We randomly selected 10 Arabidopsis *DEFL*s as reference points for validation of gene expression patterns (Table S1). Gene expression fold-change values obtained via qRT-PCR were consistent with tissue-specific and/or tissue-enhanced gene expression patterns obtained with the SBQ normalized custom array data. In most cases, fold-changes obtained from microarray data were smaller in amplitude compared with those obtained via qRT-PCR assays.

### Many Computationally Predicted Arabidopsis *DEFL*s Show Constitutive Expression Patterns

**Figure 3:**
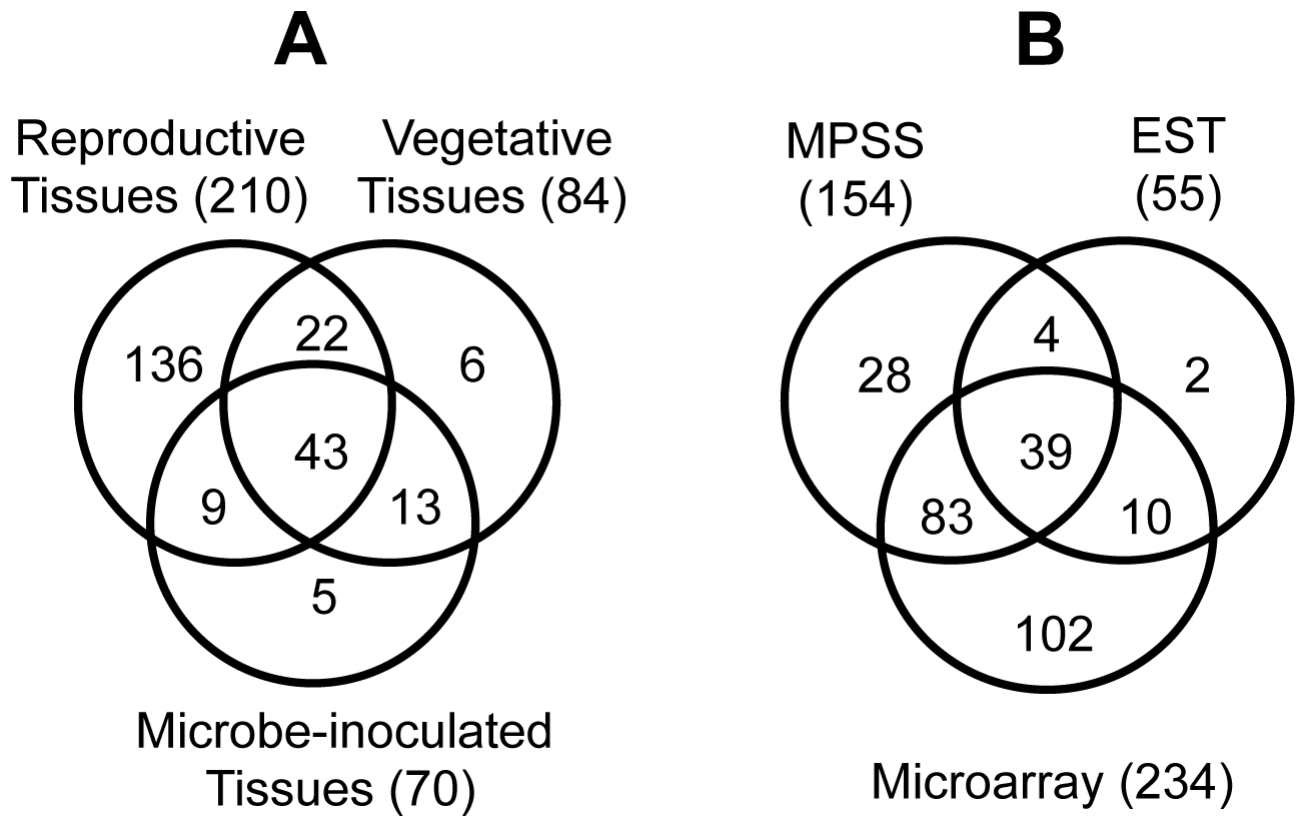
Venn diagrams of expressed Arabidopsis *DEFL*s in different treatments. A, Number of expressed *DEFL*s and *MEG*s in various tissues. B, Comparison of different gene discovery approaches for the number of identified expressed *DEFL*s by each technique.

Based on MAS5 detection call criteria [31], expression of 234 Arabidopsis *DEFL*s and *MEG*s (about 70.5% of the *DEFL*s and *MEG*s on the custom chip) was detected in one or more of the tissues examined (Figure 3A). The majority (210) were expressed in reproductive tissues (flowers and mature siliques) with a smaller number (84) expressed in vegetative tissues (roots and leaves) and pathogen-infected leaves (70) (Table 1). Approximately 43 *DEFL*s were constitutively expressed and detected in all tissues examined (Figure 3A). There were more genes expressed in reproductive tissues than in healthy and pathogen-inoculated vegetative tissues combined (Table 1). We identified *DEFL*s that were uniquely expressed in only one tissue or condition, the majority of which were unique to reproductive tissues. Arabidopsis *DEFL*s with tissue-specific expression patterns in this study are listed in Table S2.

**Table 1:** Number of Arabidopsis *DEFL* expression by tissue and condition

Organ	Number of Expressed *DEFL*s	Percent Expressed *DEFL*s
Seedlings (7- and 14-d-old)	51	16
Roots (21-d-old)	41	10
Leaves (30- to 40-d-old)	53	16
Infloresences	132	41
Siliques	141	44
Pathogen-infected leaves (30- to 40-d-old)	70	22
Total*	234	73

* Some DEFLs were expressed in more than one organ and/or condition, so, the number of expressed DEFLs in each organ/condition does not add up to the total.

To further improve the information base on the number of Arabidopsis *DEFL*s for which we have gene expression evidence, we cross-referenced the list of expressed *DEFL*s in the present study to that of gene lists reported previously by others using sequencing-based transcript profiling approaches: Massively-Parallel Signature Sequences (MPSS) database (http://mpss.udel.edu/at/) and expressed sequence tags (ESTs) in the Arabidopsis Gene Index (AGI) (http://compbio.dfci.harvard.edu/tgi/cgi-bin/tgi/gimain.pl?gudb=arab). The MPSS database is based on sequencing technology that captures very small sequence signature tags (17 bp and 20 bp) for expressed transcripts. The AGI is a culmination of sequencing efforts involving a collection of several hundred thousand ESTs. Combining data from these three approaches there are a total of 268 *DEFL*s and *MEGs* with evidence of gene expression in one or more organs and conditions (83% of the 332 *DEFLs* and *MEG*s on the custom chip) (Figure 3B). Of this total, the custom chip analysis provided new information on expression of 102 recently identified *DEFLs* in Arabidopsis that previously lacked evidence for gene expression.

### Numerous Arabidopsis *DEFL*s Show a Predominantly Reproductive Expression Pattern

**Figure 4:**
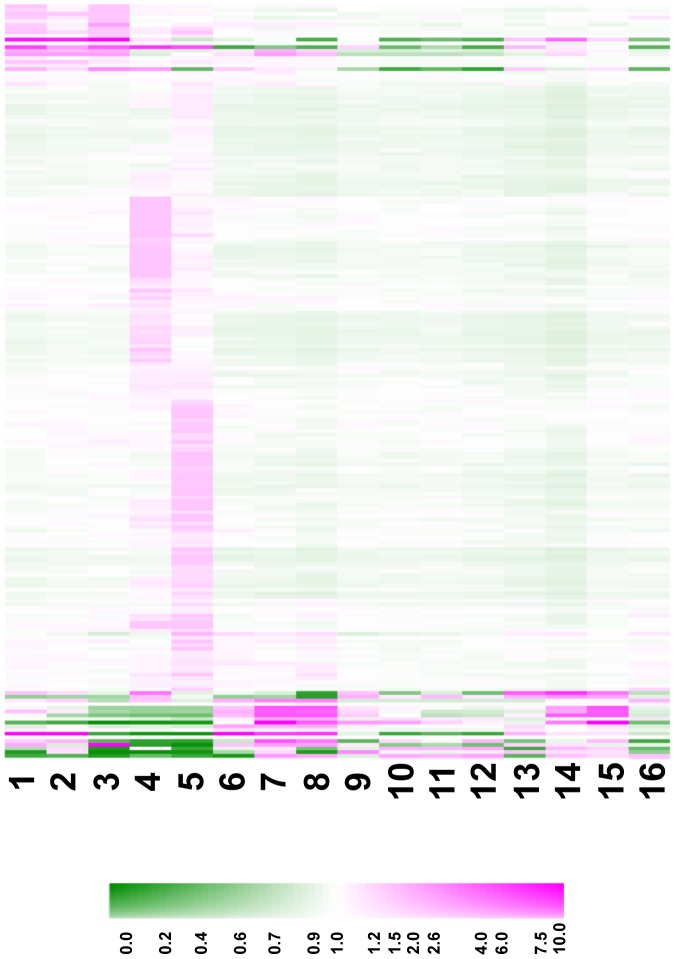
Transcript profiles of expressed Arabidopsis *DEFL*s and *MEG*s. Treatments were: (1) 7-d-old seedling, (2) 14-d-old seedling, (3) 21-d-old root, (4) inflorescences, (5) siliques, (6) Col-0 *Alternaria* mock-inoculation, (7) Col-0 *Alternaria*-inoculated leaf, (8) *dde2-2 Alternaria*-inoculated leaf, (9) *Pseudomonas* mock-inoculation at 3 hpi, (10) *Pto*DC3000 inoculated leaves at 3 hpi, (11) *Pto*DC3000*hrcC^-^* inoculated leaves at 3 hpi, (12) *Pto*DC3000*AvRpt2* inoculated leaves at 3 hpi, (13) *Pseudomonas* mock-inoculation at 9 hpi, (14) *Pto*DC3000 inoculated leaves at 9 hpi, (15) *Pto*DC3000*hrcC^-^* inoculated leaves at 9 hpi, and (16) *Pto*DC3000*AvRpt2* inoculated leaves at 9 hpi. The heat map shows median scaling of SBQ normalized signal intensity values as described in Materials and Methods. Colors represent high transcript abundance (magenta), low transcript abundance (green), and average transcript abundance (white).

The SBQ-normalized signal intensity values of *DEFLs* from a variety of Arabidopsis tissues, including healthy and pathogen-infected tissues presented in Table S2, are schematically displayed as a heat map (Figure 4). From this figure it is apparent that a majority of expressed Arabidopsis *DEFLs* showed either unique or enhanced expression in inflorescences (representing a pool of floral development stages, with the most mature at anthesis) and mature siliques at stage 17 of plant development [38], [39]. Approximately 30 *DEFL*s and *MEGs* were expressed in all Arabidopsis reproductive organs examined. A further 58 *DEFLs* and *MEGs* were uniquely expressed in mature siliques.

**Figure 5:**
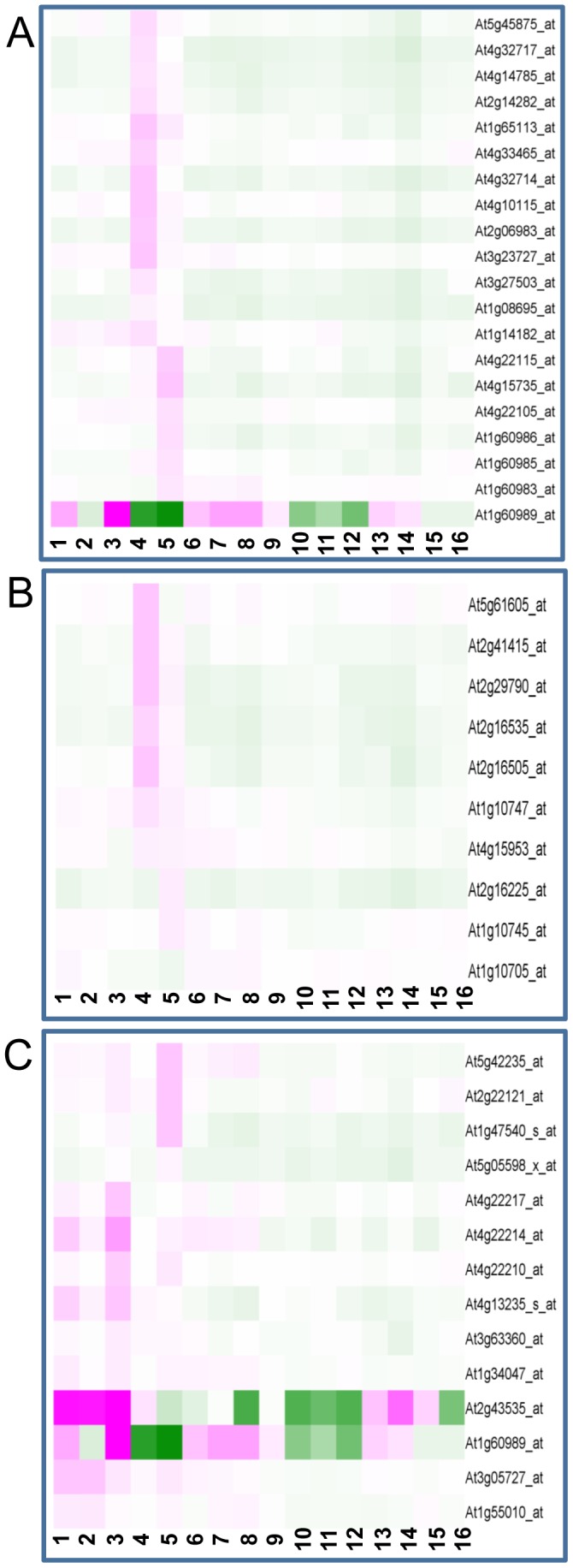
Gene expression profiles of Arabidopsis. A, *SCR*s. B, *MEG*s. C, Seeding- and root-specific/enhanced *DEFL*s. Treatments and heatmap colors are as depicted in Figure 4.

*SCRs* and *MEGs* were expected to be expressed primarily in reproductive tissues because members of these gene subgroups were known to play regulatory roles in pollen-stigma interactions [40], or in seed development, respectively [26], [41]. Many Arabidopsis *SCR*s and *MEG*s are not represented on the Affymetrix ATH1 array. By contrast, our custom array contains probe sets representing 28 *SCR*s and 15 *MEG*s. We evaluated the tissue-specific and developmentally regulated expression patterns of these *DEFL* subgroups using the microarray data collected from several tissues and organs during normal growth conditions and from pathogen-infected tissues. We found that *SCR*s were expressed primarily in inflorescence tissues, with the exception of one *SCR* (At1g60989), which showed expression in vegetative tissues that was enhanced in 21-d-old-roots (Figure 5A). Our data also show that eighteen *SCR*s are uniquely expressed in reproductive tissues (11 uniquely expressed in inflorescences, three in mature siliques, and two in both organs). Most *MEG*s were expressed uniquely in inflorescences and siliques, although four were expressed in one or more of the tissues examined (Figure 5B). Furthermore, we observed that several previously known plant defensins (*PDF*2.4, *PDF*2.6, *PDF*3.1, *PDF*3.2) are up-regulated in reproductive tissues (Figure S3A).

### Arabidopsis *DEFL* Expression in Vegetative Tissues

Expression of approximately 26% of the *DEFL*s and *MEG*s on the custom chip was detected in vegetative tissues, but only five *DEFL*s were expressed uniquely in these tissues. Interestingly, approximately 14 *DEFLs*, including an *SCR* (At1g60989) and a plant defensin (*PDF1.5*; At1g55010), were predominantly expressed in seedlings and/or roots, in a seedling- and root-specific manner (Figure 5C). Previously known plant defensins *PDF*2.1, *PDF*2.2, *PDF*2.3, and *PDF*2.5 also showed up-regulated expression in vegetative tissues primarily in seedlings and root tissues (Figure S3A).

**Figure 6:**
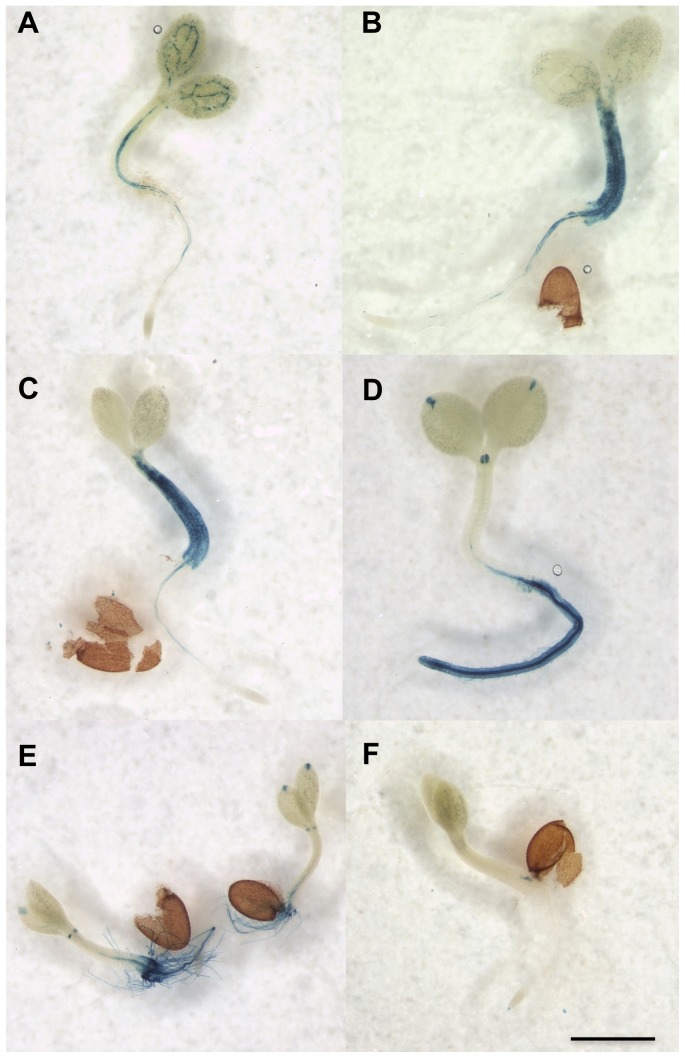
Transcriptional fusion analysis of Arabidopsis *DEFL* promoter constructs in T4 transgenic seedlings expressing pSCR:*LhG4*/*OP*:GUS. Histochemical staining for GUS activity in 1-week-old seedlings transformed with promoter constructs of Arabidopsis *DEFLs*. A, At1g60989. B, At2g26020. C, At5g60553. D, At3g63360. E, At4g30074. F, At5g05598. Scale bar 10 µm.

We further examined expression patterns of six Arabidopsis genes identified by microarray analysis using the highly sensitive *LhG4*/*OP* transactivation system [42]. Genomic regions upstream of the initiation codon were fused to *LhG4* and transformed in a pOP::GUS reporter line. We selected six genes for detailed analysis: the *SCR* expressed in roots (At1g60989), defensin *PDF*1.2b (At2g26020), which is expressed constitutively and induced in leaves by pathogen infection, and four *DEFLs*, At5g60553 (expressed in seedlings and siliques), At3g63360 (expressed in seedlings and roots), At4g30074 (expressed constitutively), and At5g05598 (expressed in roots and siliques). Histochemical analysis of 1-week-old T4 transgenic seedlings revealed that the SCR promoter is active in the vascular tissue of roots and cotyledons (Figure 6A). The *PDF*1.2b promoter is active throughout the seedling with intense GUS staining in hypocotyl tissues (Figure 6B). The hypocotyl and root is also the site of activity of the At5g60553 promoter (Figure 6C). As expected, the promoters of At3g63360 and At5g05598 were active in roots (Figure 6D,F) and the promoter of At4g30074 active throughout the seedling (Figure 6E). These transcriptional fusions confirm our microarray data, revealing that *DEFL* expression in vegetative and reproductive development is confined to discrete organs and/or cell types.

### Identification of Fungal Pathogen-Responsive Arabidopsis *DEFL*s

To identify pathogen-responsive *DEFL*s in Arabidopsis, wild type and *dde2-2* mutant plants were challenged with the necrotrophic fungal pathogen *Alternaria brassicicola*, the causal agent of early blight [43]. *DDE2* encodes allene oxide synthase, which is essential for jasmonate (JA) biosynthesis [44]. In agreement with previous findings [43], [45], *dde2-2* plants were found to be more susceptible to *A. brassicicola* than Col-0 (Figure S4). Gene expression profiles of leaf samples collected at 24 h post inoculation (hpi) and those from mock-inoculated plants were compared. A total of 21 genes including eight *DEFL*s and five *PDF*s were significantly (P<0.05) differentially expressed (Table S4). Of these, two *DEFL*s (At3g05730, At2g43550) were significantly repressed 24 hpi with *A. brassicicola*, regardless of the plant genotype. Transcript abundance of one *DEFL* (At2g43530) was also significantly reduced by *A. brassicicola* inoculation in the *dde2-2* mutant background compared with Col-0 (Table S4). Differentially regulated genes include known JA-dependent defense response genes such as *PDF1.1*/*PDF1.2*, *PDF1.2a-c*, and *PDF1.3* (At1g75830, At5g44420, At2g26020, At5g44430, At2g26010), though the probe sets for the latter three genes are complicated by potential cross-hybridization (i.e., they have ‘_x_at’ suffixes). A hevein-like protein precursor (*HEL1*; At3g04720) and a gene encoding a basic chitinase (At3g12500) were also differentially expressed. As expected, transcripts corresponding to JA-responsive marker genes did not accumulate to the same extent in the *dde2-2* mutant as they did in Col-0. However, three Arabidopsis *DEFL*s showed strong JA-dependent induction (Table S4).

### *DEFL* Expression Patterns in Arabidopsis Coincide with Bacterial Pathogen Recognition

In order to assess which *DEFL* genes are expressed during bacterial pathogen responses, we collected leaves challenged with bacterial strains *Pto*DC3000, *Pto*DC3000*AvrRpt2*, or *Pto*DC3000*hrc*C^-^ at 3 and 9 hpi. We found that at 3 hpi, 12 *DEFL*s and six *PDF*s were significantly (P<0.05) differentially regulated among the three bacterial strains used and the number of significantly (P<0.05) differentially regulated genes increased to 29 *DEFL*s and eight *PDF*s at 9 hpi (Table S5). All but two genes that were differentially regulated at 3 hpi were also differentially regulated at 9 hpi. *Pto*DC3000-induced genes at 9 hpi included 13 *DEFL*s and six *PDF*s and *Pto*DC3000-repressed genes included 16 *DEFL*s and two *PDF*s (Table S5).

Two well-studied modes of pathogen recognition mechanisms occur in plants: recognition of molecular patterns (pathogen-associated molecular patterns, PAMPs, or microbe-associated molecular patterns, MAMPs) common to microbes and recognition by host resistance proteins of certain effectors delivered inside plant cells by pathogens. Many effector proteins are known to interfere with host immune signaling. Recognition of PAMPs and type III effectors by host plants leads to induction of PAMP-triggered immunity (PTI) and effector-triggered immunity (ETI), respectively. We expected differences in transcript accumulation of PTI- and ETI-responsive genes in leaves inoculated with *Pto*DC3000*hrc*C^-^ (a strain unable to deliver effectors which induce PTI) and *Pto*DC3000*AvrRpt2* (AvrRpt2 is an effector that triggers ETI in Col-0) strains compared to plants inoculated with wild type *Pto*DC3000. By these criteria, a *DEFL* (At5g43285), five plant defensins (*PDF1.2a-c*, *PDF1.3, PDF1.4*), and several marker genes including a hevein-like protein (HEL/PR-4; At3g04720), and two high-affinity nitrate transporters (At3g45060, At1g08090) could be considered as PTI-responsive genes. Genes that showed evidence of ETI-response expression patterns included two *DEFLs* (At3g61185, At5g46871), a plant defensin (*PDF*1.1; At1g75830), as well as genes encoding pathogenesis-related protein 3 (PR-3; At3g12500), PR-1 (At2g14610), 4-coumarate:Coenzyme A ligase (4CL; At3g21230), and a MYB108 transcription factor (At3g06490) (Table S5).

### Co-expression Analysis using Known Marker Genes for Defense Signaling Pathways Verified Expression Patterns of Pathogen-Responsive *DEFL*s

We extended our analysis of the Arabidopsis custom microarray data for co-expression patterns using three marker genes for host defense responses. Genes used for the co-expression analysis include *PDF1.2a* (At5g44420), which is a marker for host defense via the JA-mediated pathway [46], [47], *coronatine-insensitive 1* (*COI1*; At2g39940), also known to be induced by JA [48], as well as the MAMPs-inducible gene, *FLG22-inducible receptor-like kinase 1* (*FRK1*; At2g19190), which is implicated in early defense signaling associated with the perception of flagellin [49]. Our analysis identified 16 genes, including four *DEFL*s, that showed co-regulated (R^2^ ≥ 0.7) expression patterns with *FRK1* (Figure S3B). Other co-regulated genes with *FRK1* included *PR-1* (At2g14610, At1g64280), *PR-5* (At1g75040), *ascorbate peroxidase* (At1g07890), *β-glucanase* (At3g57260), and *glycerol kinase* (At1g80460). We identified two *DEFL*s (At2g16367, At5g18407) and four *PDF*s (At1g19610, At2g26010, At2g26020, At5g44430) that had co-regulated (R^2^ ≥ 0.7) expression patterns with *PDF1.2a* and/or *COI1* (Figure S3C). Except for At2g16367, transcript abundance of the remaining co-regulated *DEFL*s and other genes was significantly regulated by *Pseudomonas* and/or *A. brassicicola* inoculation (Tables S4 and S5).

### Medicago *DEFL*s Show Tissue- and Condition-Specific Gene Expression Patterns

**Figure 7:**
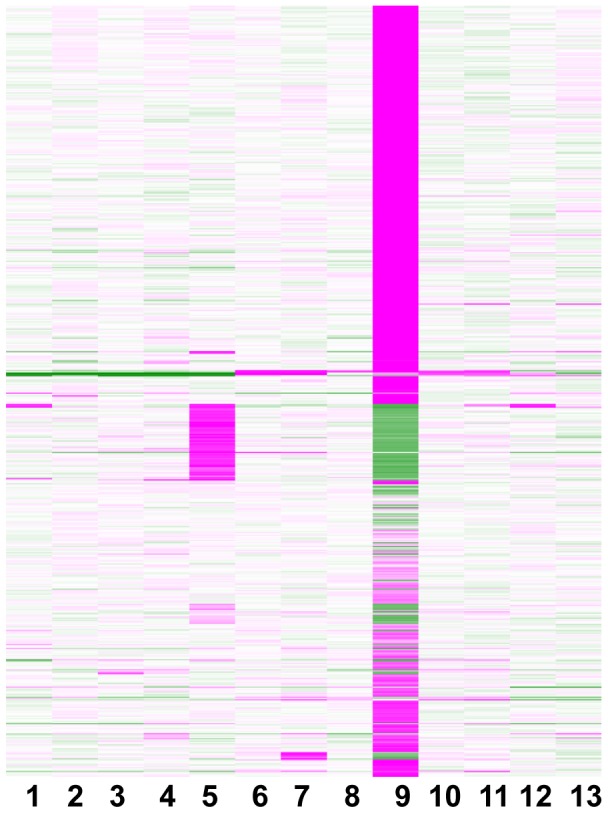
Transcript profiles of expressed Medicago *DEFLs*. Treatments were: (1) Germinating seed, (2) stem, (3) flower buds, (4) flowers, (5) seed, (6) uninoculated root for mycorrhizal control, (7) mycorrhizal root, (8) uninoculated root for nodule control, (9) nodules at 14 dpi, (10) *P. medicaginis* mock-inoculated root, (11) *P. medicaginis*-inoculated root, (12) *C. trifolii* mock-inoculated leaf, and (13) *C. trifolii*-inoculated leaf. The heat map shows median scaling of SBQ normalized signal intensity values as described in materials and methods and colors represent high transcript abundance as depicted in Figure 4.

Expression profiles of 684 Medicago *DEFL*s were surveyed in *M. truncatula* accession A17 using the MtDEFL custom chip. We profiled different plant organs as well as various tissues inoculated with plant pathogens and symbiotic microbes. The SBQ-normalized signal intensity values presented in Table S3 are schematically displayed as a heatmap in Figure 7. The most prominent gene expression patterns were very strong gene expression in either root nodules or in seeds.

**Figure 8:**
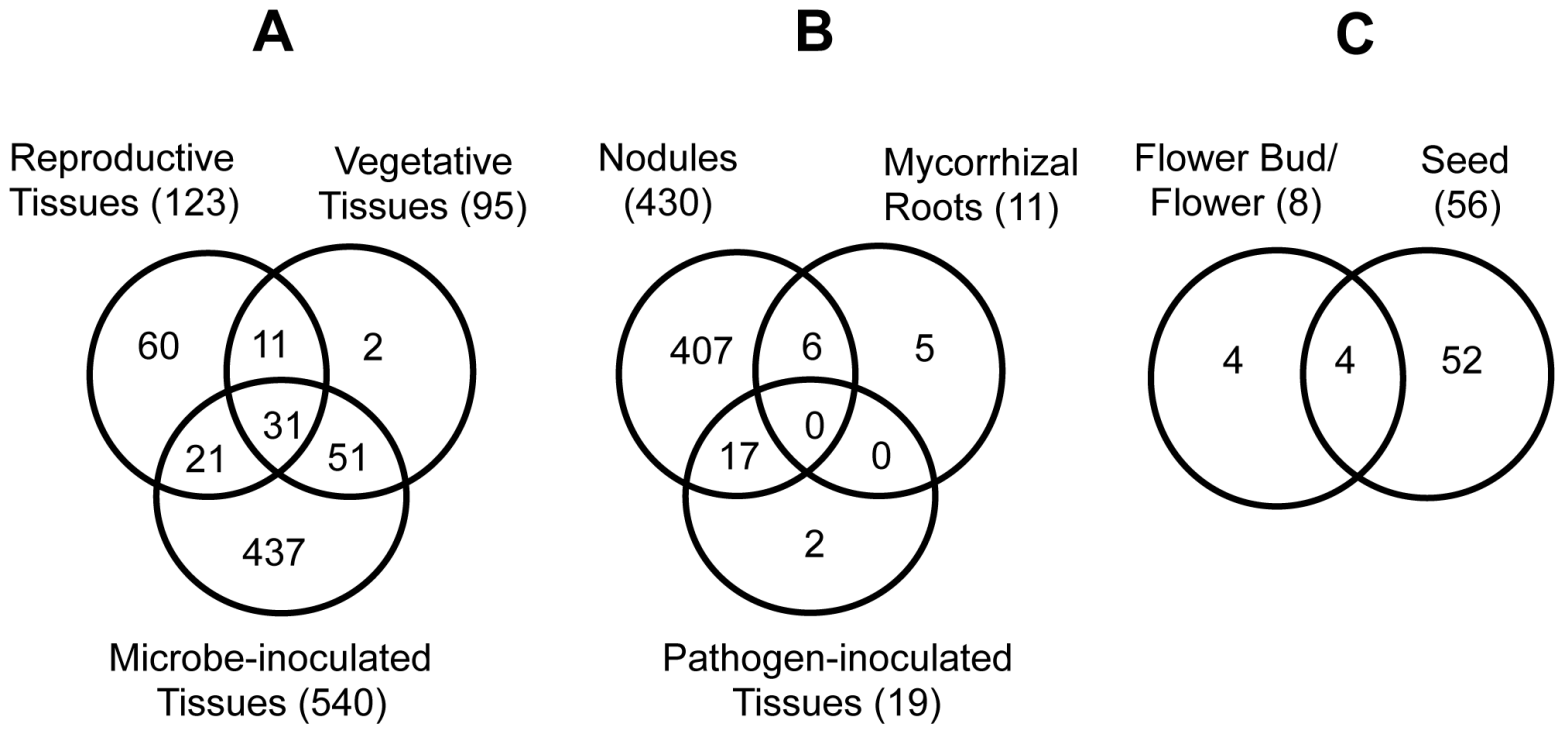
Venn diagrams of Medicago *DEFL*s expressed in different treatments. A, Comparison among all tissue types. B, Tissues inoculated with microbial symbionts and plant pathogens. C, Reproductive tissues.

Based on MAS5 gene expression detection criteria [31], a total of 176 Medicago *DEFL*s (26% of the Medicago *DEFL*s on the custom chip) were expressed in one or more of the following Medicago tissues that were grown under normal conditions (i.e. not inoculated with microbes): germinating seeds, leaves, stems, flower buds, open flowers, immature seeds, and roots (Figure 8A). In vegetative tissues 95 *DEFL*s were expressed and 123 *DEFL*s were expressed in reproductive tissues (Figure 8A). Approximately 31 *DEFL*s were expressed in all vegetative, reproductive, and microbe-inoculated tissues (Figure 8A). Approximately 79% of the total *DEFL*s on the custom chip were expressed in microbe-inoculated tissues (Figure 8A, B).

Among Medicago *DEFL*s expressed in reproductive stages, only MtgAC142396_38300, MtgCU302334_101329, MtgAC149130_78022, and MtgAC147712_80528 were uniquely expressed in flower buds and/or flowers. In contrast, 52 *DEFL*s were uniquely expressed in developing Medicago seeds (Figure 8C). Medicago *DEFL*s with tissue-specific expression patterns in this study are listed in Table S3.

### Expression of Medicago *DEFL*s in Symbiotic Tissues

Medicago *DEFL* expression during symbiotic interactions of *M. truncatula* A17 was evaluated using the widely studied arbuscular mycorrhizal (AM) fungus *Glomus intraradices* and the root nodule forming soil bacterium *Sinorhizobium meliloti* strain Sm1021. For AM symbiosis, *G. intraradices*-colonized roots (Myc+) were collected at 28 days post-inoculation (dpi), which is a heavily infected stage. Approximately 44 Medicago *DEFL*s were expressed in Myc+ roots. For statistical analysis of gene expression during AM symbiosis, signal intensity values in Myc+ colonized roots were compared with signal intensity values with non-mycorrhizal control roots (Myc−). Eleven *DEFL*s were significantly up-regulated (P<0.05), whereas one Medicago *DEFL* was significantly down-regulated (P<0.05) in Myc+ roots compared to Myc- roots (Table S6).

The majority (77%) of the *DEFL*s on the custom chip were found to be expressed in root nodules formed by *S. meliloti* at 14 dpi. Statistical analysis of SBQ normalized data revealed that 515 Medicago *DEFL*s were significantly up-regulated (P<0.05) in nitrogen-fixing root nodules compared to uninoculated root controls of similar age (Table S7). Only two Medicago *DEFLs* (MtgCR3171614, MtTC94214) were significantly down-regulated (P<0.05) in 14 dpi nodules compared to uninoculated roots of similar age (Table S7).

### Medicago *DEFL* Expression During Pathogenic Interactions

We investigated *DEFL* expression patterns during interactions of Medicago plants with a foliar pathogen, *Colletotrichum trifolii,* and a root pathogen, *Phytophthora medicaginis*. *Medicago truncatula* accession A17 is resistant to *C. trifolii* and displays a rapid hypersensitive response (HR) at 12 to 24 hpi [50]. To identify *DEFL*s whose expression is **coincident** with the HR, the MtDEFL chip was used to evaluate gene expression patterns in *M. truncatula* A17 at 24 hpi with *C. trifolii*. Approximately 36 *DEFL*s were expressed in *C. trifolii* inoculated leaf samples. Statistical analysis showed that four *DEFL*s were significantly down-regulated (P<0.05) in *C. trifolii* inoculated leaf samples compared to mock-inoculated leaf (Table S8).

The accession A17 is moderately susceptible to *P. medicaginis* with lesions being typically small and occurring about 5 dpi [51]. Our preliminary study using qRT-PCR detected the highest significant differential expression of two antimicrobial genes (*thaumatin-like protein* and *PR10*) occurred at 4 dpi with *P. medicaginis*. Expression analysis using the MtDEFL array revealed that approximately 41 *DEFL*s were expressed in infected root segments of Medicago in response to *P. medicaginis*, whereas only eight *DEFL*s were significantly up-regulated (P<0.05) in *Phytophthora*-challenged roots compared to mock-inoculated root tissues of similar age at 4 dpi (Table S9).

## Discussion

### A Small Set of *DEFL*s are Pathogen-Responsive in Arabidopsis and Medicago

*DEFL*s generally share common features in gene structure and genomic organization to defensins, which make up one subfamily within the *DEFL* superfamily [7], [8], [9]. It is expected that *DEFL*s that possess antimicrobial activity are likely to be up-regulated in response to pathogens. Thus, to identify additional *DEFL*s with possible antimicrobial activity, we profiled *DEFL* expression in Arabidopsis plants infected with bacterial or fungal pathogens as well as in infected Medicago roots and leaves. Through expression profiling and co-expression analysis with known marker genes for early defense signaling and JA-mediated host response, we identified several pathogen-responsive Arabidopsis *DEFL*s. In contrast, few *DEFL*s were expressed in Medicago roots infected with *P. medicaginis* or in leaves exhibiting an HR in response to *C. trifolii*. These genes comprise a subset of *DEFL*s that warrant further investigation into their antimicrobial activity and role in plant defense.

Plants activate numerous defense responses upon recognition of invading pathogens. Several plant defensins with demonstrated protease inhibitor or antimicrobial activity are induced by pathogens via the JA signaling pathway [4], [46], [47]. Of the 15 plant defensins previously described, we found that *PDF1.1*, *PDF1.2a-c*, *PDF1.3,* and *PDF1.4* were induced by pathogens and showed co-regulated expression patterns with known marker genes in pathogen-infected leaves. Transcript abundance of *PDF2.1*, *PDF2.2*, *PDF2.3,* and *PDF2.5* were down-regulated by pathogen infection, but showed higher transcript abundance in seedlings, roots, and inflorescence tissues. The expression profiles of these plant defensins were consistent with previous reports that showed up-regulated expression of *PDF1.2* and down-regulated expression of *PDF2.2* by infection with *A. brassicicola* [4].

Resistance against necrotrophic pathogens such as *A. brassicicola* requires JA-mediated signaling, leading to activation of defenses including defensins [43], [45]. Results of experiments using *A. brassicicola* infections of the *dde2-2* mutant, with impaired JA synthesis and increased susceptibility to *A. brassicicola*, are highly suggestive that expression of some *DEFL*s are induced via JA-mediated signaling. Co-expression of these genes with known JA-signaling marker genes (*PDF1.2a*, *COI1*) used in our analysis suggested that a few of the novel *DEFL*s are involved in plant defense and their expression is likely mediated by the JA-signaling defense pathway.

Complex transcriptional reprogramming involving a large number of genes has been shown to be involved during PTI and ETI. Gram-negative bacterial pathogens such as the hemibiotroph *Pto*DC3000 employ the type III secretion system to translocate effector proteins into the host cytoplasm. The bacterial HrcC protein is an essential component of the type III secretion system, so bacteria with the *hrc*C mutation cannot deliver type III effectors into the host cytoplasm. Therefore, the strain *Pto*DC3000*hrc*C^-^ can induce PTI but cannot interfere with PTI, whereas the wild type *Pto*DC3000 induces and interferes with PTI. Consequently, *Pto*DC3000 reproduces well in Col-0 but *Pto*DC3000*hrc*C^-^ cannot. On the other hand, the strain *Pto*DC3000*AvrRpt2* induces ETI because Col-0 expresses the resistance protein RPS2, which recognizes AvrRpt2. We used *Pto*DC3000*hrcC^-^* and *Pto*DC3000*AvrRpt2* to characterize *DEFL* transcriptional responses in Col-0 leaves during PTI and ETI, respectively. In total, we identified 39 Arabidopsis *DEFL*s (Table S4) that were differentially regulated in response to *P. syringae*. We also found that considerably more *DEFL*s were expressed at 9 hpi compared to either 3 hpi with *P. syringae* or 24 hpi with *A*. *brassicicola* (37 *DEFL*s compared to three *DEFL*s and 13 *DEFL*s, respectively). Our data suggest that several *DEFL*s may be important in PTI and/or ETI. Furthermore, co-expression analysis using the PAMPs-induced marker gene, *FRK1*, suggested that the expression of up to nine *DEFL*s may be related to early defense signaling in response to *P. syringae*.

### Putative Roles for *DEFL*s in Reproduction and Symbiotic Microbial Associations

More than 100 *DEFL*s are specifically expressed in Arabidopsis inflorescences and/or siliques while approximately 60 *DEFL*s are expressed in reproductive tissues in Medicago, most of which (56 *DEFL*s) are expressed only in seeds. The results in Arabidopsis were consistent with earlier observations of gene expression patterns of some known *DEFL*s [8], [52], [53] that were expressed solely in siliques [52] or ovules [53] and detected via MPSS or whole-genome tiling arrays. The specific expression of Arabidopsis *DEFL*s in synergids, which were found to be important in the origin of pollen tube attractant, suggest that some of those Arabidopisis *DEFLs* are likely to be involved in different aspects of fertilization [54]. Medicago *DEFL* expression patterns in this study were consistent with previously observed patterns of gene expression in several EST libraries and defensin gene expression *i*n *situ* [55], [56]. In particular, we observed that *MtDef1.1*, *MtDef2.1*, and *MtDef4.6* were expressed at high levels in seeds and *MtDef4.2* showed high constitutive expression in all tissues examined. A few of the Medicago defensins including the probe sets MtTC101060_x_at, MtTC104515_at, MtAL385826_s_at, MtTC105086_at, and MtTC98064_at were found to be the most significantly expressed genes in response to *G. intraradices* root colonization. Previously, TC77480, corresponding to MtTC101060_x_at in the custom chip, was reported to be strongly expressed in *M. truncatula* roots at 2 to 4 weeks post-inoculation with *G. versiforme* [55]. The recent report that over-expression of MtDef4.2 in transgenic Arabidopsis results in enhanced disease resistance [56] suggests that some Medicago *DEFL*s play a role in plant defense.

*DEFL*s have a variety of properties that make them ideal molecular signals, especially in regulatory control of fertilization. They exist in large tandemly duplicated gene families with high birth and death rates that include numerous pseudogenes [9]. Thus, new genes with novel specificities can be created quickly via gene conversion events. Additionally, they are highly polymorphic and rapidly evolving [9], [19], [57]. *DEFL*s have been co-opted for the purpose of preventing self-pollination in the Brassicaceae [58] in which the defensin-like SP11 (also known as SCR) was shown to bind to the S-locus receptor kinase to initiate a cascade of events leading to self-pollen rejection. In *Torenia fournieri*, a pair of *DEFL*s was shown to serve as the elusive molecular signal exuded from the female gametophyte that attracts pollen tubes to the embryo sac [21]. *DEFL*s have also been co-opted to erect species barriers in animals [59].

From MtDEFL array analysis and previous *in silico* analysis of Medicago ESTs it was observed that a large number of Medicago *DEFL*s are expressed almost exclusively in nitrogen-fixing nodules [7], [11]. A recently published report regarding a few *DEFL*s found in nodules (called nodule-specific cysteine-rich peptides in that study) indicated that nodule DEFL peptides similar to some defensins triggered membrane modifications and inhibited cytokinesis in bacteria [17]. In addition, ectopic expression of selected nodule-specific cysteine-rich peptides in *Lotus japonicus*, a legume that lacks DEFL peptides, triggered terminal differentiation of *Rhizobium loti* in nodules. Based on these findings and additional reports of fungal hyphal branching on application of defensin peptides [60], it is possible that the *DEFL*s might induce similar changes in mycorrhizal hyphae. To further characterize the nodule *DEFL*s, we have performed in-depth examinations of the expression patterns of nodule *DEFL*s, using different symbiotic bacterial mutants and developmentally arrested nodules (Nallu et al., unpublished).

## Conclusion

Here we show that a custom microarray analysis provides a rich source of information on the expression patterns of the large family of *DEFL*s in two model plant systems for which expression data was lacking. Our study revealed distinct temporal and spatial *DEFL* expression in Arabidopsis and Medicago plants. We cataloged *DEFL*s that are constitutively expressed and *DEFL*s that are expressed uniquely in reproductive tissues, seeds, seedlings, roots, or leaves. A small group of *DEFL*s was identified that are uniquely induced in response to pathogens. The patterns of gene expression revealed notable differences in *DEFL* expression specificity in the two species. Arabidopsis and Medicago are sister clades within the dicot subclass Rosidae. Medicago belongs to the Fabaceae, or legume family, and its genome lacks extensive macrosynteny with Arabidopsis [61]. The custom microarray results reveal that most Arabidopsis *DEFL*s are expressed in reproductive tissues, whereas in Medicago the majority of *DEFL*s are expressed in nitrogen-fixing nodules. The large number of *DEFL*s expressed in seeds is the commonality across the two plant species. These results raise an intriguing question about the evolution of *DEFL*s in dicots and their diverse functions. One of the possible functions of Arabidopsis *DEFL*s in inflorescences might be to prevent self-pollination and/or cross-species hybridizations. On the other hand, the rapid expansion of *DEFL*s in Medicago exclusively expressed in root nodules is indicative of extensive selection to support the biological process of nitrogen fixation. Further functional analysis on the *DEFL* subfamilies found in Arabidopsis and Medicago will aid our understanding their diverse functional roles of *DEFLs* in plants. In addition, comparative genomic studies will add a new perspective on the evolution of this prolific and highly variable gene family among flowering plants.

## Materials and Methods

### AtMtDEFL Array Design Features and Description

The AtMtDEFL array was commissioned from Affymetrix (Santa Clara, CA) and includes one probe set each for 317 Arabidopsis *DEFL*s and 15 *DEFL*-related *MEG* family members [8], [30]. It also includes 684 probe sets (some probe sets exactly matched an entire subfamily of related *DEFL*s) representing all 756 Medicago *DEFL*s that were previously identified [8]. The custom chip also includes probe sets of 31 Arabidopsis genes to serve as markers for expression in particular morphological structures and/or biological processes (e.g., *PR-1* through *PR-5*, *ABI1*, *EIN2*, seed- and floral-specific genes). There were also 23 Medicago probe sets that were expected to be specific to seeds, other morphological structures, or to pathogen induction, based on the MtDB2 query interface [62]. In addition, the custom chip included 171 probe sets directly from the Arabidopsis ATH1 array (Affymetrix), which we have designated as ‘invariant genes’. These probe sets were chosen because they had a coefficient of variation (CV) of less than 10% across a collection of more than 5,000 AtGeneExpress array hybridizations (http://arabidopsis.org/info/expression/ATGenExpress.jsp).

Similarly, 172 invariant probe sets were taken directly from the Affymetrix Medicago Genome Array, because they showed less than 10% CV across more than 100 GeneChip hybridizations [63]. Beyond these core invariants, 60 Arabidopsis probe sets were also included that had a CV of less than 3% across select tissue subsets. However, 20 Medicago invariant probe sets, chosen based on the sparse set of experimental conditions available at the time of custom chip design, failed the 10% CV threshold, when their expression profiles were re-analyzed against the 100 Medicago hybridizations available after the custom chip was produced. Probe sets for marker genes and invariant genes were designed exactly as they appeared on the standard ATH1 and Medicago Genome arrays.

Probe sets from the two species are interspersed on the array although separate chip description files (cdfs) were created at Affymetrix with the intent that hybridization and analysis of the array would be performed using only one species at a time. All probe sets were represented by 11 perfect match and 11 single mismatch probes, as was typical of Affymetrix array designs. As with all Affymetrix designs, a probe set is given an ‘_x_at’ suffix to indicate that one or more probes within the probe set cross-hybridizes to an unintended target and an ‘_s_at’ suffix is used to indicate that all 11 probes in the probe set exactly match two or three gene family members. In general, expression values for cross-hybridizing probe sets (i.e., those with ‘_x_at’ suffixes) from Arabidopsis should be interpreted with caution. For Medicago, the ‘_s_at’ and ‘_x_at’ suffixes ascribed to probe sets at the time of array design are misleading because at that time, the Medicago genome was unassembled and consisted of highly redundant, partially assembled BAC sequences. We have remapped all probes to the current release (Mt3.5v5) of the genome annotation (see Text S1, Tables S10 and S11) and determined that only 11% of Medicago probe sets cross-hybridize to unintended genes and only 7% exactly match two or three gene family members.

### Arabidopsis Plant Material and Growth Conditions

Col-0 and *dde2-2* (At5g42650) in the Col-0 background [44] were used in this study. Seeds were imbibed and stored at 4°C for 3 d before planting in an appropriate growth medium. Arabidopsis plants used for harvesting inflorescences (-2 to 0 d post-anthesis), siliques at stage 17 of plant development [38], [39], and pathogen-inoculated leaves were grown in SUNGRO sunshine potting mix (SUNGRO Horticulture, Pine Bluff, AZ). For harvesting root tissues, Arabidopsis plants were grown in vermiculite (SUNGRO Horticulture). Seeds for seedling tissue harvest were sterilized by treating with 10% (v/v) bleach for 20 min, rinsed in sterile water several times and kept at 4°C for 3 d before planting on rectangular plastic plates containing 1x Murashige and Skoog (MS) salt mixture supplemented with 1% sucrose in 1% agar. Plates were kept vertical in a growth chamber and seedlings were collected 7 and 14 d after sowing. All Arabidopsis experiments were conducted in growth chambers set at a light intensity of approximately 170 µmol m^−2^ s^−1^, temperature of 22°C, and a 12-h photoperiod. Plants were watered with tap water as needed. Three biological replicates were produced for each treatment.

### Pathogen Infection of Arabidopsis Plants

*A. brassicicola* strain ATCC96866 was cultured on 0.5X potato dextrose agar at 22°C. Spores were harvested from 10-d-old cultures by washing with water and filtering through four layers of cheesecloth. Spore concentrations were determined using a hemocytometer. For pathogen challenge, leaves of 21-d-old Arabidopsis plants were inoculated with *A. brassicicola* spores according to Nafisi et al. [64]. Briefly, the third, fourth, and fifth true leaves of each plant were inoculated for monitoring disease symptoms as well as for microarray analysis of *DEF*L expression. The three inoculated leaves from each plant were collected and bulked for RNA isolation. The mock-inoculated plants were treated in a similar manner with sterile water containing 0.02% Tween-20 used as inoculum. Leaf samples were collected 24 h after inoculation and were immediately frozen in liquid nitrogen for storage at−80°C until needed for total RNA extraction.

For experiments with bacterial pathogens, virulent *P. syringae* pv. *tomato* DC3000 (*Pto*DC3000), a derivative of *Pto*DC3000 lacking *hrc*C (*Pto* DC3000*hrc*C^-^), and *Pto*DC3000 carrying *AvrRpt*2 (*Pto*DC300*AvrRpt2*) were cultured as described previously [65], [66], [67]. Four-week-old Arabidopsis plants were inoculated with bacterial strains essentially as described before [67]. Control plants were mock-inoculated with 5 mM MgSO_4_. Leaves were collected 3 and 9 hpi, immediately frozen in liquid nitrogen, and stored at −80°C until needed for total RNA extraction.

### Medicago Plant Culture and Tissue Harvest

Seeds of *M. truncatula* accession A17 were sterilized and germinated as described previously [68]. Unless indicated otherwise, Medicago plants were grown from 24 h germinated seedlings transplanted into six-inch pots containing an equal mixture of Turface (Profile Products LLC, Buffalo Grove, IL) and Metro Mix (SUNGRO - 200 series) and fertilized with Osmocote PLUS (15-9-12). All Medicago experiments were conducted in controlled growth chambers with a 16 h photoperiod, 200 to 300 µmol m^−2^ s^−1^ light intensity, 50% relative humidity, and 25°C and 21°C day- and night-time temperatures, respectively. All Medicago tissues and organs were collected from three biological replicates, with samples collected and pooled from multiple plants in each replicate. Plant samples were immediately frozen in liquid nitrogen during harvest and were stored at −80°C for subsequent RNA extraction.

Medicago flower buds that were 6 to 8 mm (measured from the base of the bud excluding the petiole to the tip of the sepals), tightly closed, and whose petals had not emerged beyond the sepals were collected from 80-d-old plants. Closed flowers (with petals bigger than the sepals) and open flowers were harvested from 80-d-old plants and pooled. For seed samples, flowers were tagged at anthesis as described by Wang and Grusak [69] and a pool of seeds were collected 10 to 21 d after pollination from 98-d-old plants. Seedlings with radicles 8 to 12 mm long were collected at 24 h post-germination. Stem segments consisting of the fifth and sixth internodes from the shoot apex were collected from 8-week-old Medicago plants as described previously [70].

To obtain nodule tissues, plants were grown on buffered nodulation medium [71], pH 6.5, with 1.2% plant tissue culture grade agar (Sigma-Aldrich, St. Louis, MO) in 245 mm×245 mm plates (Corning, Lowell, MA, USA). The radicles of sterile germinated seeds were placed on a moist, sterile germination paper on top of the agar medium and plates were wrapped with a sterile black cotton cloth (Cotton Club Black, #074300603820, Wal-Mart). Plates were maintained vertically in a growth chamber as described above. At 5 d after planting, plants were inoculated with 100 µL/root of a washed suspension of *S. meliloti* strain Sm1021 [OD600] =  0.05 in sterile water). Control roots were mock inoculated with 100 µL sterile water. At 14 dpi, approximately 5-cm-long nodule-bearing root segments were harvested from inoculated plants. At the same time, roots consisting of approximately 5-cm root segments corresponding to the regions harvested for 14 dpi nodules were collected from mock-inoculated plants. Root tips were removed from all plants at the time of harvest to reduce transcript contribution from root meristematic cells.

For mycorrhizal roots, germinated seedlings were transplanted into Turface (Profile Products LLC) and were grown in growth chambers. *Glomus intraradices* spores were prepared from plates as previously described [72]. Twenty-one–day-old plants were inoculated with 1,000 *G. intraradices* spores per plant as described previously [73]. Control plants were mock inoculated with distilled water. At 28 dpi, mycorrhizal roots without the root tip were harvested and immediately frozen in liquid nitrogen. Roots of mock-inoculated plants were similarly harvested. Throughout the experiment, plants were fertilized with 100 mL/pot using half-strength Hoagland's solution with 1x nitrogen, as needed.

For *Phytophthora*-infected roots, germinated seeds were placed in CYG seed germination pouches (Mega International, West St. Paul, MN), grown, and inoculated as described previously [51]. At 4 dpi, infected hypocotyl and root segments were harvested from both the infected and mock-inoculated plants, frozen immediately in liquid nitrogen, and held at −80°C for subsequent RNA extraction.

For *Colletotrichum*-infected leaves, 19-d-old plants were sprayed to run-off with a suspension of *C. trifolii* spores at 10^6^ spores/mL containing 0.05% Tween-20 as described previously [51]. Mock-inoculated plants were sprayed with sterile water containing 0.05% Tween-20. After 24 h, leaves from pathogen-inoculated or mock-inoculated plants were collected, frozen immediately in liquid nitrogen, and held at −80°C for subsequent RNA extraction.

### Generation of Arabidopsis Lines Using the *LhG4* transactivation System and GUS Histochemical Staining

Genomic regions corresponding to promoter regions upstream of the initiation codon of selected Arabidopsis *DEFL*s were cloned by PCR using template genomic DNA isolated from rosette leaves and primer sequences (Table S12) that contained attB2 and attB1 sites upstream of the 5′ and 3′ gene-specific priming sequences, respectively. PCR products were cloned into the pGEM T-easy vector following the manufacturer's instructions (Promega, Madison, WI). The resulting plasmid DNA containing promoter fragments was subjected to BP Clonase recombination reaction with pDONR 207 (Invitrogen, Carlsbad, CA) and transformed into *E. coli* DH5α. Cloned promoter sequences were validated by sequencing. Transcriptional fusion of *DEFL* promoters were constructed by transferring the cloned promoter regions in front of the chimeric *LhG4* [42], and transferred into the pBINPLUS binary vector using standard molecular biology protocols [74]. Binary vectors were mobilized into *Agrobacterium tumefaciens* strain GV3101. The resulting *A. tumefaciens* strains were used for floral dip transformation of Arabidopsis plants carrying an pOp:GUS reporter construct for transactivation [75]. Transgenic plants were selected on MS medium supplemented with 50 mg/mL kanamycin. Twenty transformed lines carrying a single copy of the transgene were selected for each construct.

For histochemical analysis, 1-week-old seedlings were vacuum-infiltrated with a staining solution containing 0.5 mM 5-bromo-4-chloro-3-indolyl-β-D-glucuronic acid, 0.15 M NaH_2_PO_4_, pH 7, 2 mM K_3_Fe(CN)_6_, 2 mM K_4_Fe(CN)_6_, and 0.05% Triton X-100. Seedlings were incubated overnight in the staining solution and cleared overnight in 100% ethanol.

### RNA Extraction and GeneChip Hybridization

Approximately 150 mg of Arabidopsis or Medicago tissue ground in liquid nitrogen was used for total RNA extraction. Total RNA from Medicago seeds and germinating seedlings was extracted using the Spectrum Plant Total RNA Kit (Sigma-Aldrich) following the manufacturer's instructions. Total RNA from mature Arabidopsis siliques was extracted using the hot phenol total RNA extraction protocol [76]. Total RNA from all other Arabidopsis and Medicago tissues was extracted using TRIZOL reagent (Invitrogen) following the manufacturer’s instructions. During RNA extraction, contaminating genomic DNA was removed by incubating samples with TURBO™ DNase following standard procedures suggested by the supplier (Applied Biosystems, Foster City, CA). The integrity and quality of total RNA was verified using the Agilent 2100 Bioanalyzer RNA 6000 Nano LabChip (Agilent Technologies, Santa Clara, CA). For Arabidopsis siliques, 100 ng total RNA was labeled using a 2-cycle Affy chip labeling protocol for eukaryotic reactions following the manufacturer’s suggested procedures (Affymetrix). For the other Arabidopsis tissues and all Medicago samples, 10 µg of total RNA was used to produce biotin-labeled cRNA using Affymetrix suggested procedures for 1-cycle eukaryotic reactions (Affymetrix). Ten micrograms of biotin-labeled cRNA, fragmented as suggested by Affymetrix, was hybridized to the AtMtDEFL array. The integrity and quality of labeled and fragmented biotin-labeled cRNA was verified using the Agilent 2100 Bioanalyzer RNA 6000 Nano LabChip (Agilent Technologies). GeneChips were hybridized, washed, stained and scanned as previously described [70].

### Microarray Data Normalization and Analysis

Microarray normalizations and initial analyses were performed in R using custom scripts that made use of Bioconductor routines [77]. All scripts are available upon request. In addition to RMA [33], three microarray data normalization methods were compared. To implement the Stable-genes Based Quantile (SBQ) normalization [36], we first combined expression data from all 11 probes within a probe set into a single expression measure using the Bioconductor expression command using RMA-style background correction and median polish probe summarization. These summarized values were output from R and used as input to the SBQ perl script [36]. For RMA with Median Absent Probe set Scaling (RMAPS) we performed RMA separately on each group of biological replicates (within which the density distribution assumptions hold) using the RMA routine within the Bioconductor package. We then rescaled the log_2_ expression levels for each biological replicate group such that the median values among the set of probe sets that had an absent call (MAS5 calls routine in the affy package) across all sampled conditions were equal from one replicate group to the next. For RMA with Invariant Median Scaling (RIMS), we performed RMA separately on each group of biological replicates as above. Then we rescaled the expression levels for each replicate group according to the median value of the set of invariant genes.

We also calculated a detection call (‘present’ vs. ‘absent’) for each probe set using default MAS5 parameters except changing α_1_ = 0.05 as suggested for 11 probe pairs per probe set by Liu et al. [31]. For statistical analysis, SBQ normalized signal intensity values were log_2_ transformed and statistical analysis was based on a t-test or ANOVA (P<0.05) with Benjamini and Hochberg false discovery rate multiple testing correction [78], and the corrected p values were designated as q values. To identify *DEFL*s and marker genes that showed co-expression patterns, Pearson correlation coefficients were calculated for each probe set using the GeneSpring Expression analysis platform version 7.3 (Agilent Technologies). For gene expression data clustering analysis, the average linkage clustering algorithm and the Pearson correlation similarity measure were applied as provided in GeneSpring Expression analysis software version 7.3 (Agilent Technologies). Heat maps were generated with GeneSpring Expression analysis platform as well as the heatmap2 Bioconductor package.

All microarray data in this study have been deposited in the Gene Expression Omnibus under platform number GSE34401 (http://www.ncbi.nlm.nih.gov/geo/query/acc.cgi?acc=GSE34401) with two subseries: GSE34398 (expression data of *DEFL*s from different parts and treatments of Arabidopsis) and GSE34311 (expression data of *DEFL*s from different parts and treatments of Medicago).

### Real-time RT-PCR Assays

We employed quantitative reverse-transcription polymerase chain reaction (qRT-PCR) to validate the *DEFL* microarray data trends that were collected from several Arabidopsis tissues and pathogen challenged leaves. Initially, we evaluated the expression patterns and detection calls of candidate invariant genes included in the custom chip. Based on the microarray data collected using the AtMtDEFL chip, we selected 10 of the invariant genes for further evaluation of gene expression data stability. Genes were selected to cover a range of signal intensity values. The raw signal intensity values were imported into geNorm software [37] for gene expression stability analysis. Based on their stability score, we then selected four reference genes, *metallothionein 2B* (At5g02380), *yellow-leaf-specific gene 8* (At5g08290), *sumo conjugating enzyme 1* (At3g57870), and *polyubiquitin* (*UBQ11*), for validation of gene expression stability using qRT-PCR assays. Gene-specific primers (Table S12) were designed using PrimerExpress software (Applied Biosystems). Gene expression stability analysis was performed using the average threshold cycle (Ct) of the four candidate reference genes and the geNorm software. Total RNA extraction procedures were as described above and first-strand cDNA was prepared from 2 µg of total RNA with the Superscript RT II kit (Invitrogen) and oligo dT primers (Sigma-Aldrich) at 200 ng/reaction, according to the manufacturer's instructions. RT-PCR conditions were as described previously [79].

## Supporting Information

Figure S1 **Scatter plots of normalized gene expression values obtained from three boutique array methods as compared to whole-array RMA across 36 ATH1 arrays.** A, SBQ vs RMA. B, RMAPS vs RMA. C, RIMS vs RMA. All three boutique array normalizations used only a subset of 299 probe sets that correspond to genes represented on the AtMtDEFL array (37 DEFLs, 171 invariants, 91 marker genes). Each was compared to the reference RMA normalization, which included all 22,810 probe sets on the Affymetrix ATH1 array in the normalization process. Expression values were log_2_ transformed. All 37 probe sets on the ATH1 array that matched an Arabidopsis *DEFL* with at least 6 of 11 exact-match probes per probe set were included in the analysis. GEO accessions for ATH1 arrays included three biological replicates of: GSE1491 (seedlings), GSE5630 (cotyledons, leaves, senescent leaves), GSE5631 (roots), GSE5632 (carpels, stage 9 flowers, stamens), GSE5633 (stems), GSE5634 (old and young siliques), and GSE7227 (seeds).
(TIF)

Figure S2 **geNorm ranking of expression stability of candidate Arabidopsis reference genes for use in qRT-PCR analysis.** A, Expression stability was calculated using the custom chip microarray data. B, Expression stability for selected genes using qRT-PCR data. Refer to Materials and Methods for experimental details.
(TIF)

Figure S3 **Gene expression profiles of Arabidopsis** (A) defensins, (B) genes co-regulated with *receptor-like protein kinase* (*FRK1;* At2g19190) involved in early defense signaling, and (C) genes co-regulated with JA marker genes *PDF1.2a* (At5g44420) and/or *coronatine-insensitive 1* (*COI1;* At2g39940). Treatments were: (1) 7-d-old seedling, (2) 14-d-old seedling, (3) 21-d-old root, (4) inflorescences, (5) siliques, (6) Col-0 *Alternaria* mock-inoculation, (7) Col-0 *Alternaria*-inoculated leaf, (8) *dde2-2 Alternaria*-inoculated leaf, (9) *Pseudomonas* mock-inoculation at 3 hpi, (10) *Pto*DC3000 inoculated leaves at 3 hpi, (11) *Pto*DC3000*hrcC^-^* inoculated leaves at 3 hpi, (12) *Pto*DC3000 *AvRpt2* inoculated leaves at 3 hpi, (13) *Pseudomonas* mock-inoculation at 9 hpi, (14) *Pto*DC3000 inoculated leaves at 9 hpi, (15) *Pto*DC3000*hrcC^-^* inoculated leaves at 9 hpi, and (16) *Pto*DC3000 *AvRpt2* inoculated leaves at 9 hpi. The heat map shows median scaling of SBQ normalized signal intensity values as described in Materials and Methods. Colors represent high transcript abundance (magenta), low transcript abundance (green), and average transcript abundance (white) as depicted in Figure 4.
(TIF)

Figure S4 **Enhanced susceptibility of** ***dde2-2*** **to** ***Alternaria brassicicola*** **(strain ATCC 96866) 4 days after inoculation.** The third, fourth, and fifth true leaves of 21-d-old plants were inoculated by placing 10 µL droplets of water containing 10^5^ spores/mL on the adaxial surfaces of the leaves. In wild type plants, necrosis is confined to the area of the inoculum, while chlorotic and water-soaked lesions spread beyond the inoculation droplet in *dde2-2*.
(TIF)

Figure S5 **Raw log_2_ signal intensity distribution plots for AtDEFL array experiments profiling roots, leaves, siliques, and inflorescences.** The three different color lines represent independent biological replicates. The large peak in these plots at low intensity values typically represents absent probe sets in the noise-level. Systematic shifts in these plots will be reduced or eliminated by most normalization methods, but changes in shape of the curve may reflect real biological differences among the samples.
(TIF)

Figure S6 **Raw log_2_ signal intensity distribution plots for Affymetrix ATH1 array experiments profiling roots, leaves young siliques, and flowers.** The three different color lines represent independent biological replicates. The distributions in these plots are much broader and less variable than the custom array, since more than 22,000 genes are represented. Nevertheless, some variations in peak height and shape are evident among different plant organs. GEO accessions represented: GSM131558-60 (roots), GSM131498-500 (leaves), GSM131685-7 (siliques), GSM131576-8 (flowers).
(TIF)

Figure S7 **Heatmaps of Arabidospsis** ***DEFL*** **absolute expression after applying four different normalization algorithms.** A, RMA using all 22,810 probe sets on the Affymetrix Arabidopsis ATH1 array. B, SBQ. C, RMAPS. D, RIMS. Normalizations B to D used only a subset of 299 probe sets (37 DEFLs, 171 invariants, 91 marker genes). Systematic intensity shifts for some arrays using the RIMS method in D are highlighted with arrows. Expression values have been log_2_ transformed. Two-dimensional hierarchical agglomerative clustering was applied to the set of 37 *DEFL*s (rows) and 36 arrays (columns) from the RMA-normalized data in A. This ordering of rows and columns was preserved for panels B to D. All 37 probe sets on the ATH1 array that matched an Arabidopsis *DEFL* with at least six of 11 probe sets were included in the analysis. The 36 arrays included three biological replicates for a wide variety of morphological structures and conditions obtained from GEO: GSE1491 (seedlings), GSE5630 (cotyledons, leaves, senescent leaves), GSE5631 (roots), GSE5632 (carpels, stage 9 flowers, stamens), GSE5633 (stems), GSE5634 (old and young siliques), and GSE7227 (seeds).
(TIF)

Figure S8 **Heatmaps of Arabidopsis marker gene absolute expression after applying four different normalization algorithms.** A, RMA using all 22,810 probe sets on the Affymetrix Arabidopsis ATH1 array. B, SBQ. C, RMAPS. D, RIMS. Normalizations B to D used only a subset of 299 probe sets (37 DEFLs, 171 invariants, 91 marker genes). Expression values have been log_2_ transformed. Two-dimensional hierarchical agglomerative clustering was applied to the set of 91 marker genes (rows) and 36 arrays (columns) from the RMA-normalized data in A. This ordering of rows and columns was preserved for panels B to D. All 91 marker gene probe sets on the ATH1 array are exactly identical to their counterparts on the AtDEFL array. The 36 arrays are the same as those described in Figure S3.
(TIF)

Figure S9 **Heatmaps of Arabidopsis invariant gene absolute expression after applying four different normalization algorithms.** A, RMA using all 22,810 probe sets on the Affymetrix Arabidopsis ATH1 array. B, SBQ. C, RMAPS. D, RIMS. Normalizations B to D used only a subset of 299 probe sets (37 DEFLs, 171 invariants, 91 marker genes). Expression values have been log_2_ transformed. Two-dimensional hierarchical agglomerative clustering was applied to the set of 37 DEFLs (rows) and 36 arrays (columns) from the RMA-normalized data in A. This ordering of rows and columns was preserved for panels B to D. All 171 invariant gene probe sets on the ATH1 array are exactly identical to their counterparts on the AtDEFL array. The 36 arrays are the same as those described in Figure S3.
(TIF)

Figure S10 **Raw log_2_ signal intensity distribution plots for MtDEFL array experiments profiling leaves, roots, mycorrhizal roots, and root nodules.** The three different color lines represent independent biological replicates. The large peak in these plots at low intensity values typically represents absent probe sets in the noise-level. Systematic shifts in these plots will be reduced or eliminated by most normalization methods, but changes in shape of the curve may reflect real biological differences among the samples.
(TIF)

Figure S11 **Scatter plots showing correlation of normalized expression values obtained from three boutique array methods as compared to whole-array RMA across 62 MtDEFL arrays.** A, SBQ vs. RMA. B, RMAPS vs. RMA. C, RIMS vs. RMA. All three boutique array normalizations used only a subset of 565 probe sets that correspond to genes represented on the MtDEFL array (370 DEFLs - black circles, 172 invariants - green plus signs, 23 marker genes - blue crosses). Each was compared to the reference RMA normalization, which included all 61,278 probe sets on the Affymetrix Medicago array in the normalization process. Expression values have been log_2_ transformed. All 370 probe sets on the Medicago array that matched a Medicago *DEFL* with at least six of 11 probe sets were included in the analysis. The 62 arrays included three biological replicates for a wide variety of tissues and conditions obtained from ArrayExpress and GEO: E-MEXP-1097 (flowers, leaves, nodules at various developmental stages, petioles, pods, roots, seeds at various developmental stages, stems and vegetative buds), E-MEXP-1092 (methyl-jasmonate and yeast-elicited cell suspensions and controls at multiple time points), GSE8131 (NAA and BAP-treated leaf explants), and GSE8115 (roots).
(TIF)

Table S1 **qRT-PCR verification of microarray data using selected Arabidopsis genes.**
(XLSX)

Table S2 **Arabidopsis** ***DEFL*** **s custom chip SBQ normalized data.**
(XLSX)

Table S3 **Medicago** ***DEFL*** **s custom chip SBQ normalized data.**
(XLSX)

Table S4 **Arabidopsis genes differentially expressed (p<0.05) in response to** ***A*** **.** ***brassicicola***
(XLSX)

Table S5 **Arabidopsis genes differentially (p<0.05) expressed following inoculations with several** ***P. syringae*** **pv.** ***tomato*** **strains.**
(XLSX)

Table S6 **List of significantly (p<0.05) expressed Medicago genes following mycorrhizal colonization.**
(XLSX)

Table S7 **List of significantly (p<0.05) expressed Medicago genes following** ***S. meliloti*** **inoculation.**
(XLSX)

Table S8 **List of significantly (p<0.05) expressed Medicago genes at 24 hpi with** ***C. trifolii.***
(XLSX)

Table S9 **List of differentially expressed (p<0.05) Medicago genes in** ***P. medicaginis*** **-inoculated roots.**
(XLSX)

Table S10 **MtDEFL probe set mapping to Medicago Mt3.5v5 gene annotation.**
(XLSX)

Table S11 **MtDEFL individual probe mapping to Medicago Mt3.5v5 gene annotation.**
(XLSX)

Table S12 **Primer sequences of Arabidopsis genes used in this study.**
(XLSX)

Text S1 **Development of the custom AtMtDEFL array and robust data normalization methods.** Mapping Medicago probe sets to the latest annotation of the Medicago gene annotation.
(DOCX)
