# Supplementary figures and images for: Spatio-Temporal Expression Patterns of Arabidopsis thaliana and Medicago truncatula Defensin-Like Genes
Source: PLoS One. 2013 Mar 18;8(3):e58992. doi: 10.1371/journal.pone.0058992 (PMC3601123; doi:10.1371/journal.pone.0058992)

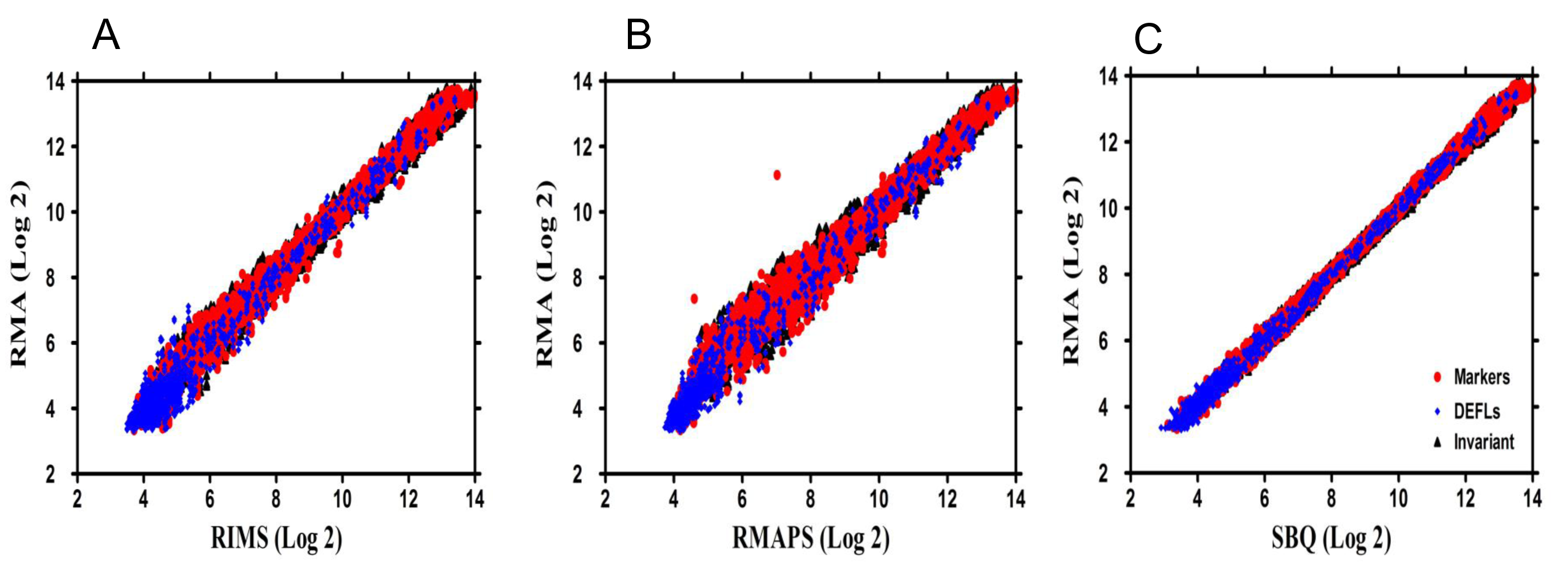

Supplement: Figure S1 — Scatter plots of normalized gene expression values obtained from three boutique array methods as compared to whole-array RMA across 36 ATH1 arrays. A, SBQ vs RMA. B, RMAPS vs RMA. C, RIMS vs RMA. All three boutique array normalizations used only a subset of 299 probe sets that correspond to genes represented on the AtMtDEFL array (37 DEFLs, 171 invariants, 91 marker genes). Each was compared to the reference RMA normalization, which included all 22,810 probe sets on the Affymetrix ATH1 array in the normalization process. Expression values were log2 transformed. All 37 probe sets on the ATH1 array that matched an Arabidopsis DEFL with at least 6 of 11 exact-match probes per probe set were included in the analysis. GEO accessions for ATH1 arrays included three biological replicates of: GSE1491 (seedlings), GSE5630 (cotyledons, leaves, senescent leaves), GSE5631 (roots), GSE5632 (carpels, stage 9 flowers, stamens), GSE5633 (stems), GSE5634 (old and young siliques), and GSE7227 (seeds). (TIF) [file pone.0058992.s001.tif]

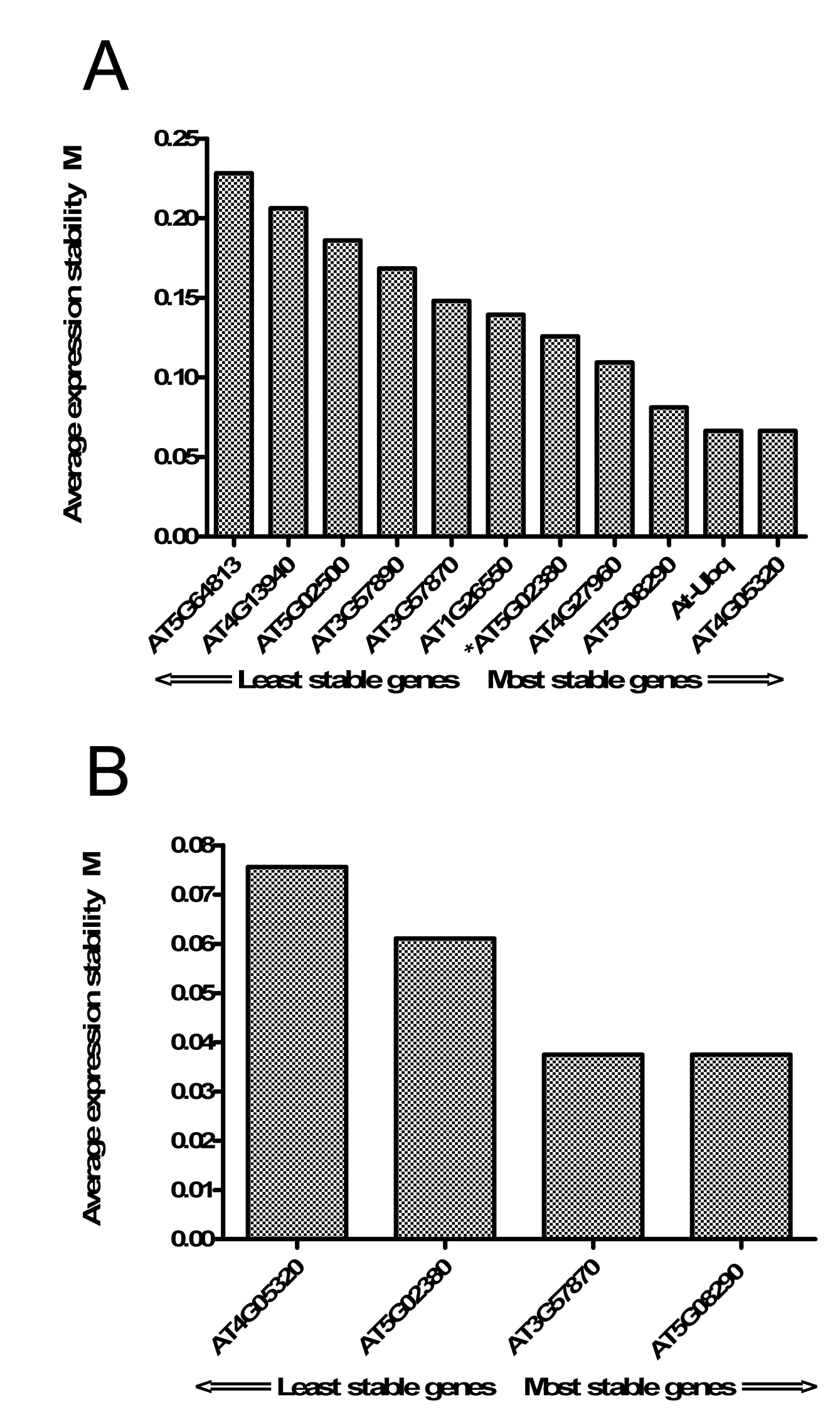

Supplement: Figure S2 — geNorm ranking of expression stability of candidate Arabidopsis reference genes for use in qRT-PCR analysis. A, Expression stability was calculated using the custom chip microarray data. B, Expression stability for selected genes using qRT-PCR data. Refer to Materials and Methods for experimental details. (TIF) [file pone.0058992.s002.tif]

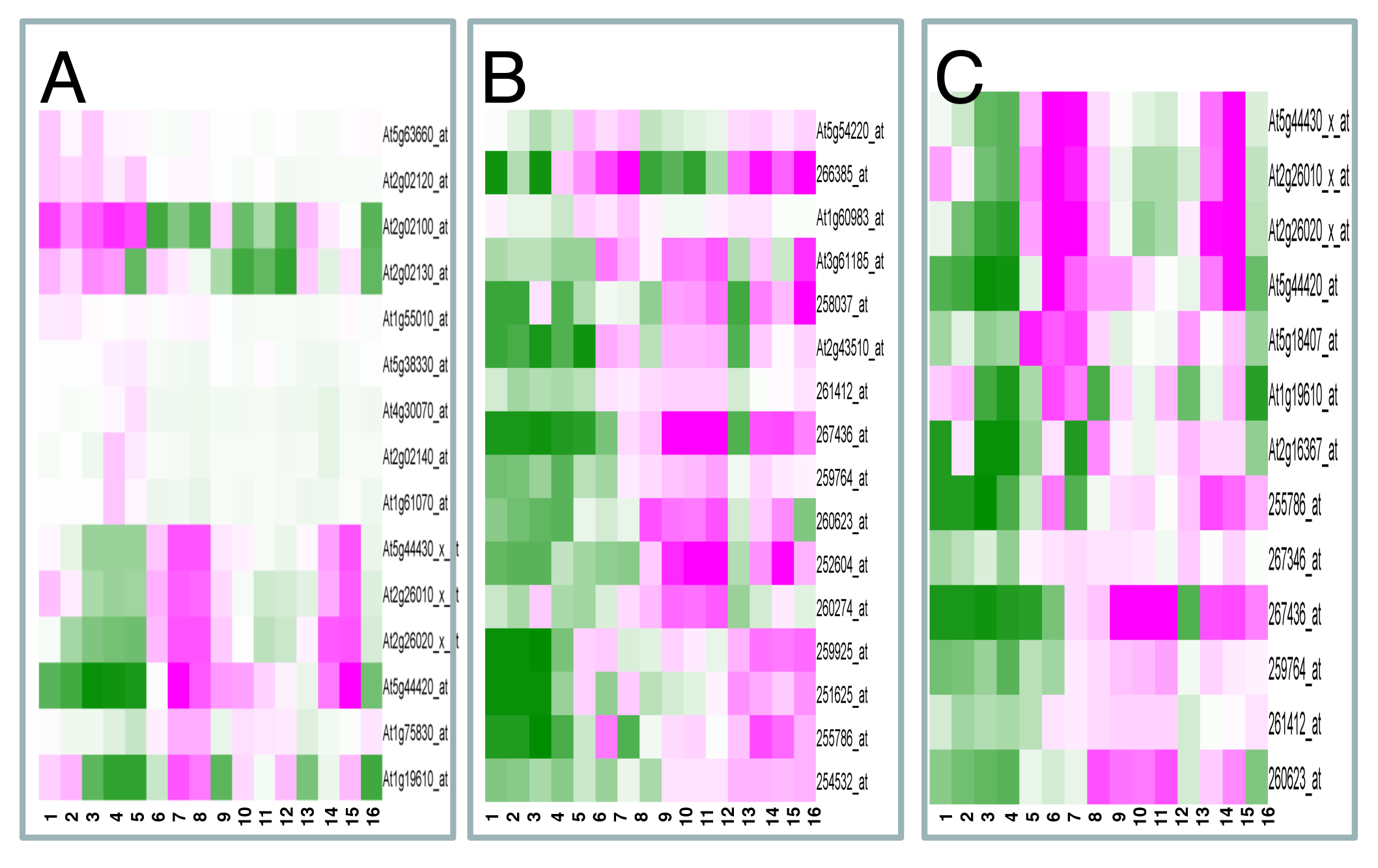

Supplement: Figure S3 — Gene expression profiles of Arabidopsis (A) defensins, (B) genes co-regulated with receptor-like protein kinase (FRK1; At2g19190) involved in early defense signaling, and (C) genes co-regulated with JA marker genes PDF1.2a (At5g44420) and/or coronatine-insensitive 1 (COI1; At2g39940). Treatments were: (1) 7-d-old seedling, (2) 14-d-old seedling, (3) 21-d-old root, (4) inflorescences, (5) siliques, (6) Col-0 Alternaria mock-inoculation, (7) Col-0 Alternaria-inoculated leaf, (8) dde2-2 Alternaria-inoculated leaf, (9) Pseudomonas mock-inoculation at 3 hpi, (10) PtoDC3000 inoculated leaves at 3 hpi, (11) PtoDC3000hrcC- inoculated leaves at 3 hpi, (12) PtoDC3000 AvRpt2 inoculated leaves at 3 hpi, (13) Pseudomonas mock-inoculation at 9 hpi, (14) PtoDC3000 inoculated leaves at 9 hpi, (15) PtoDC3000hrcC- inoculated leaves at 9 hpi, and (16) PtoDC3000 AvRpt2 inoculated leaves at 9 hpi. The heat map shows median scaling of SBQ normalized signal intensity values as described in Materials and Methods. Colors represent high transcript abundance (magenta), low transcript abundance (green), and average transcript abundance (white) as depicted in Figure 4. (TIF) [file pone.0058992.s003.tif]

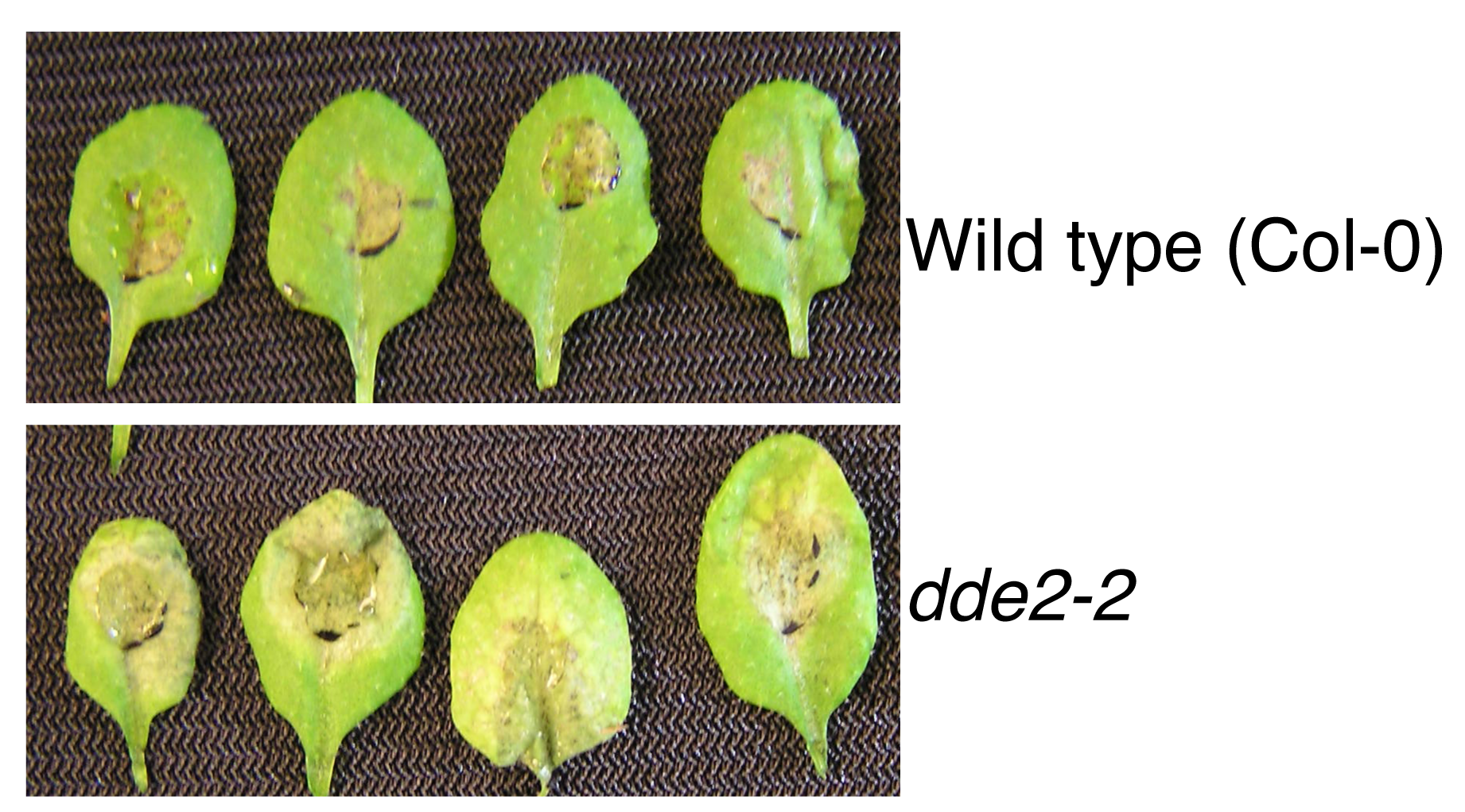

Supplement: Figure S4 — Enhanced susceptibility of dde2-2 to Alternaria brassicicola (strain ATCC 96866) 4 days after inoculation. The third, fourth, and fifth true leaves of 21-d-old plants were inoculated by placing 10 µL droplets of water containing 105 spores/mL on the adaxial surfaces of the leaves. In wild type plants, necrosis is confined to the area of the inoculum, while chlorotic and water-soaked lesions spread beyond the inoculation droplet in dde2-2. (TIF) [file pone.0058992.s004.tif]

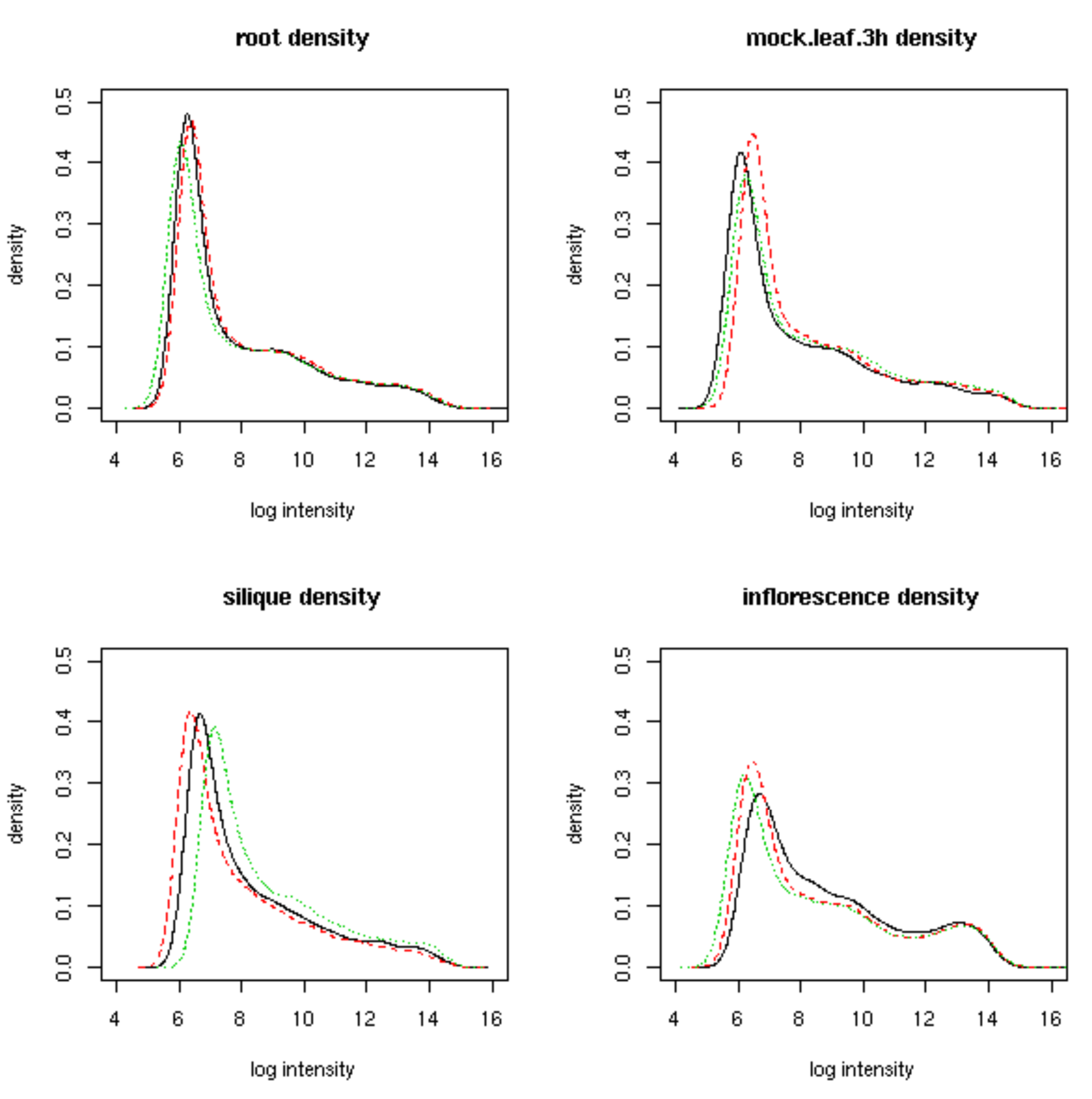

Supplement: Figure S5 — Raw log2 signal intensity distribution plots for AtDEFL array experiments profiling roots, leaves, siliques, and inflorescences. The three different color lines represent independent biological replicates. The large peak in these plots at low intensity values typically represents absent probe sets in the noise-level. Systematic shifts in these plots will be reduced or eliminated by most normalization methods, but changes in shape of the curve may reflect real biological differences among the samples. (TIF) [file pone.0058992.s005.tif]

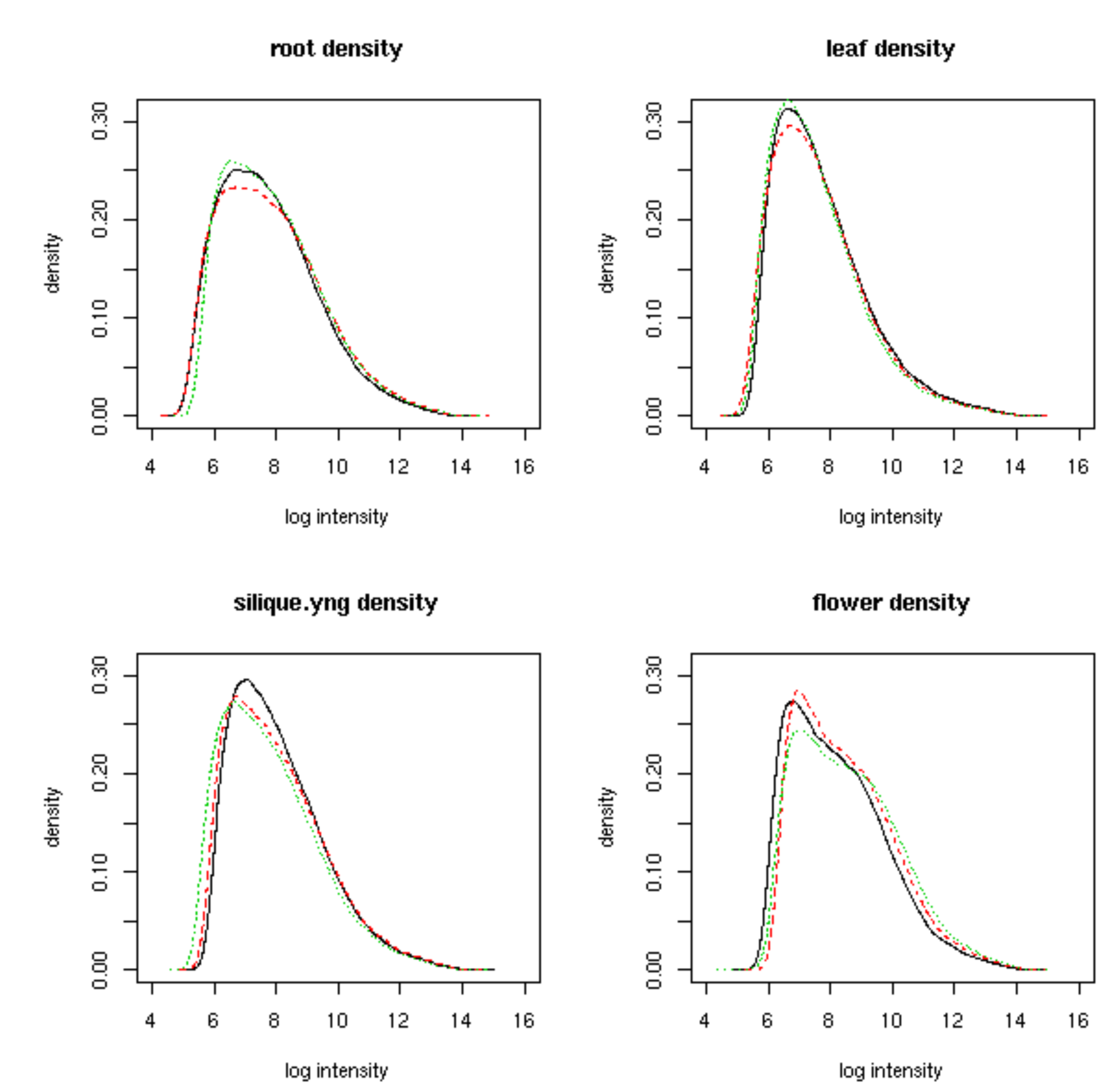

Supplement: Figure S6 — Raw log2 signal intensity distribution plots for Affymetrix ATH1 array experiments profiling roots, leaves young siliques, and flowers. The three different color lines represent independent biological replicates. The distributions in these plots are much broader and less variable than the custom array, since more than 22,000 genes are represented. Nevertheless, some variations in peak height and shape are evident among different plant organs. GEO accessions represented: GSM131558-60 (roots), GSM131498-500 (leaves), GSM131685-7 (siliques), GSM131576-8 (flowers). (TIF) [file pone.0058992.s006.tif]

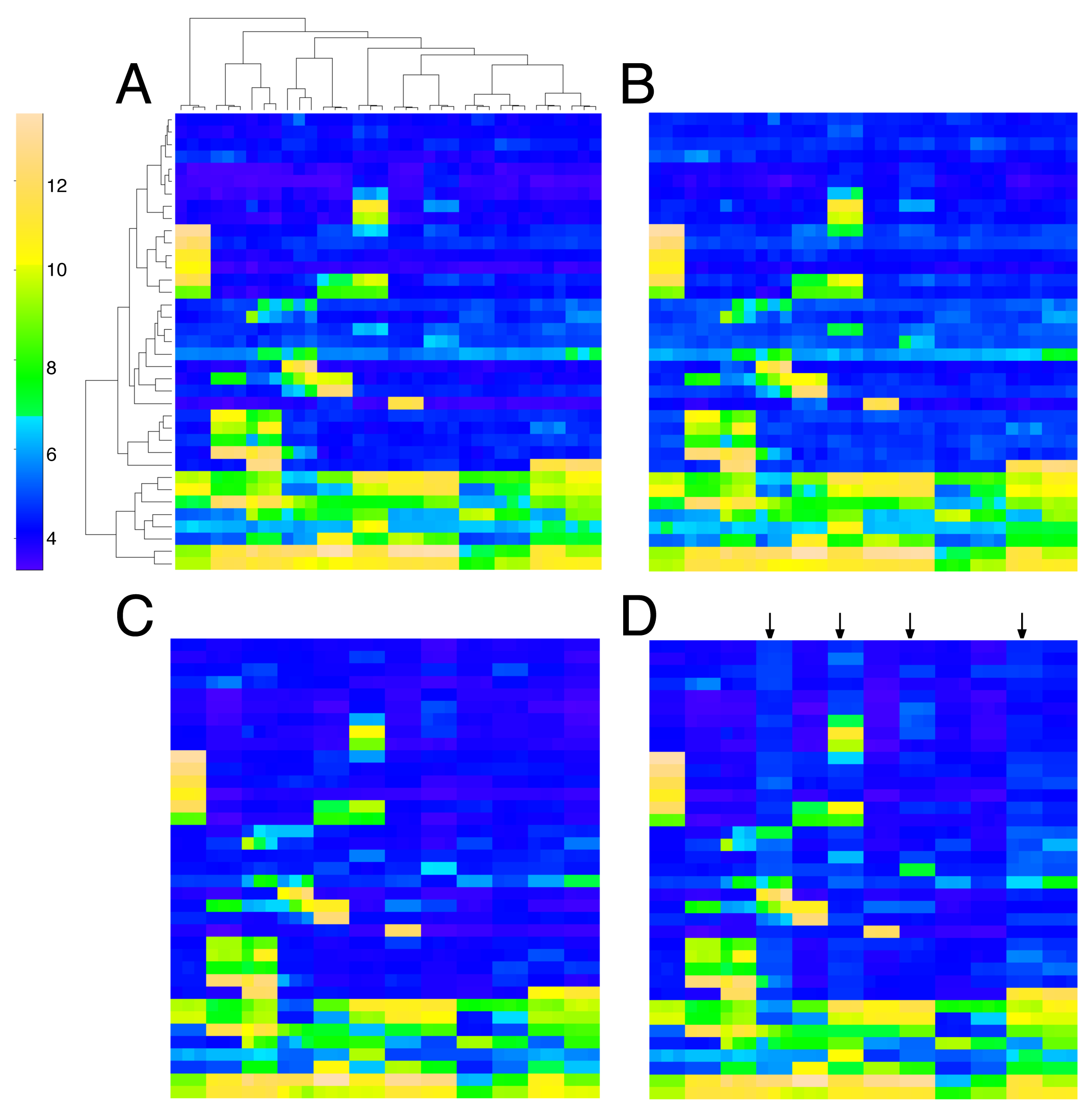

Supplement: Figure S7 — Heatmaps of Arabidospsis DEFL absolute expression after applying four different normalization algorithms. A, RMA using all 22,810 probe sets on the Affymetrix Arabidopsis ATH1 array. B, SBQ. C, RMAPS. D, RIMS. Normalizations B to D used only a subset of 299 probe sets (37 DEFLs, 171 invariants, 91 marker genes). Systematic intensity shifts for some arrays using the RIMS method in D are highlighted with arrows. Expression values have been log2 transformed. Two-dimensional hierarchical agglomerative clustering was applied to the set of 37 DEFLs (rows) and 36 arrays (columns) from the RMA-normalized data in A. This ordering of rows and columns was preserved for panels B to D. All 37 probe sets on the ATH1 array that matched an Arabidopsis DEFL with at least six of 11 probe sets were included in the analysis. The 36 arrays included three biological replicates for a wide variety of morphological structures and conditions obtained from GEO: GSE1491 (seedlings), GSE5630 (cotyledons, leaves, senescent leaves), GSE5631 (roots), GSE5632 (carpels, stage 9 flowers, stamens), GSE5633 (stems), GSE5634 (old and young siliques), and GSE7227 (seeds). (TIF) [file pone.0058992.s007.tif]

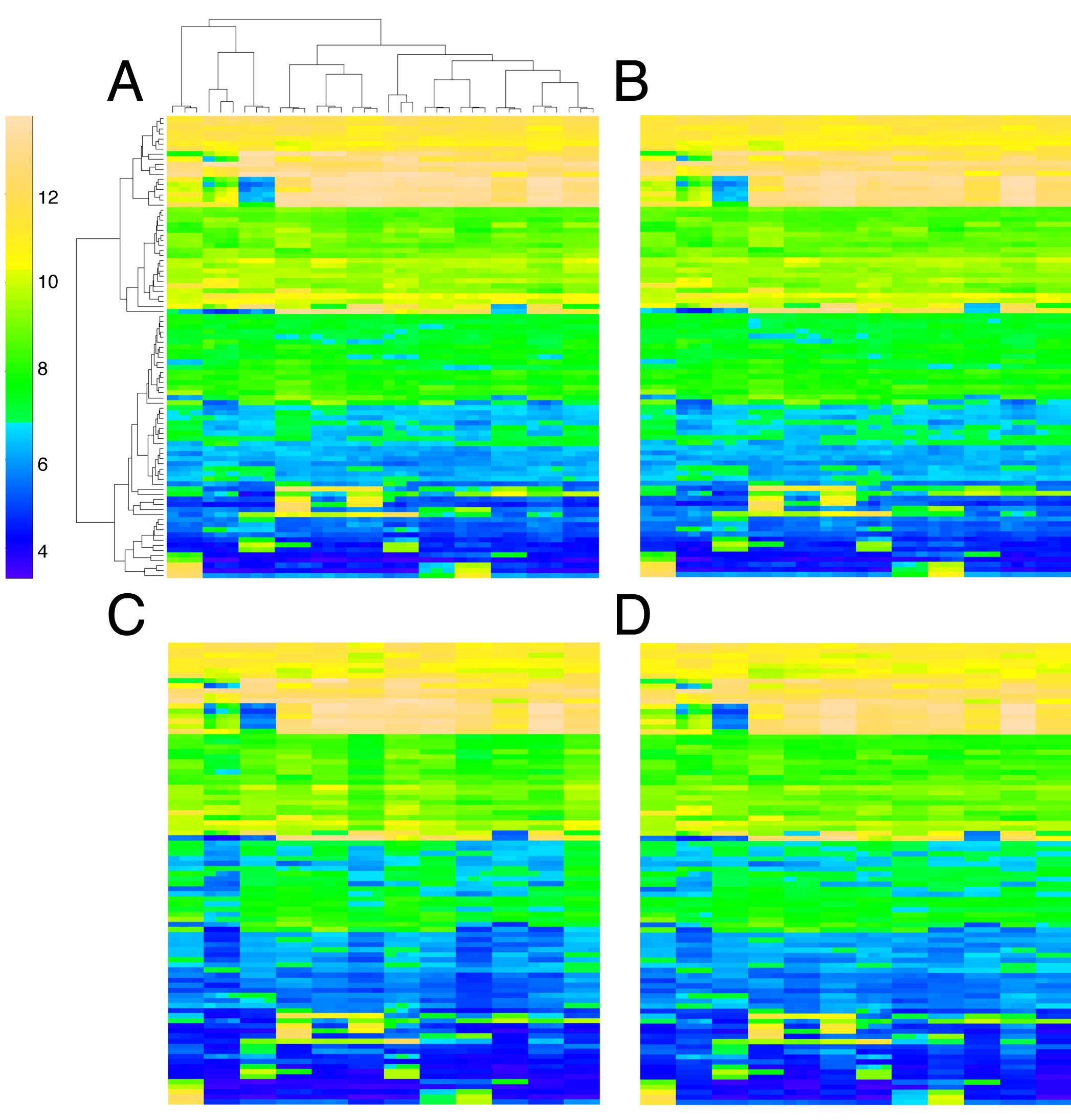

Supplement: Figure S8 — Heatmaps of Arabidopsis marker gene absolute expression after applying four different normalization algorithms. A, RMA using all 22,810 probe sets on the Affymetrix Arabidopsis ATH1 array. B, SBQ. C, RMAPS. D, RIMS. Normalizations B to D used only a subset of 299 probe sets (37 DEFLs, 171 invariants, 91 marker genes). Expression values have been log2 transformed. Two-dimensional hierarchical agglomerative clustering was applied to the set of 91 marker genes (rows) and 36 arrays (columns) from the RMA-normalized data in A. This ordering of rows and columns was preserved for panels B to D. All 91 marker gene probe sets on the ATH1 array are exactly identical to their counterparts on the AtDEFL array. The 36 arrays are the same as those described in Figure S3. (TIF) [file pone.0058992.s008.tif]

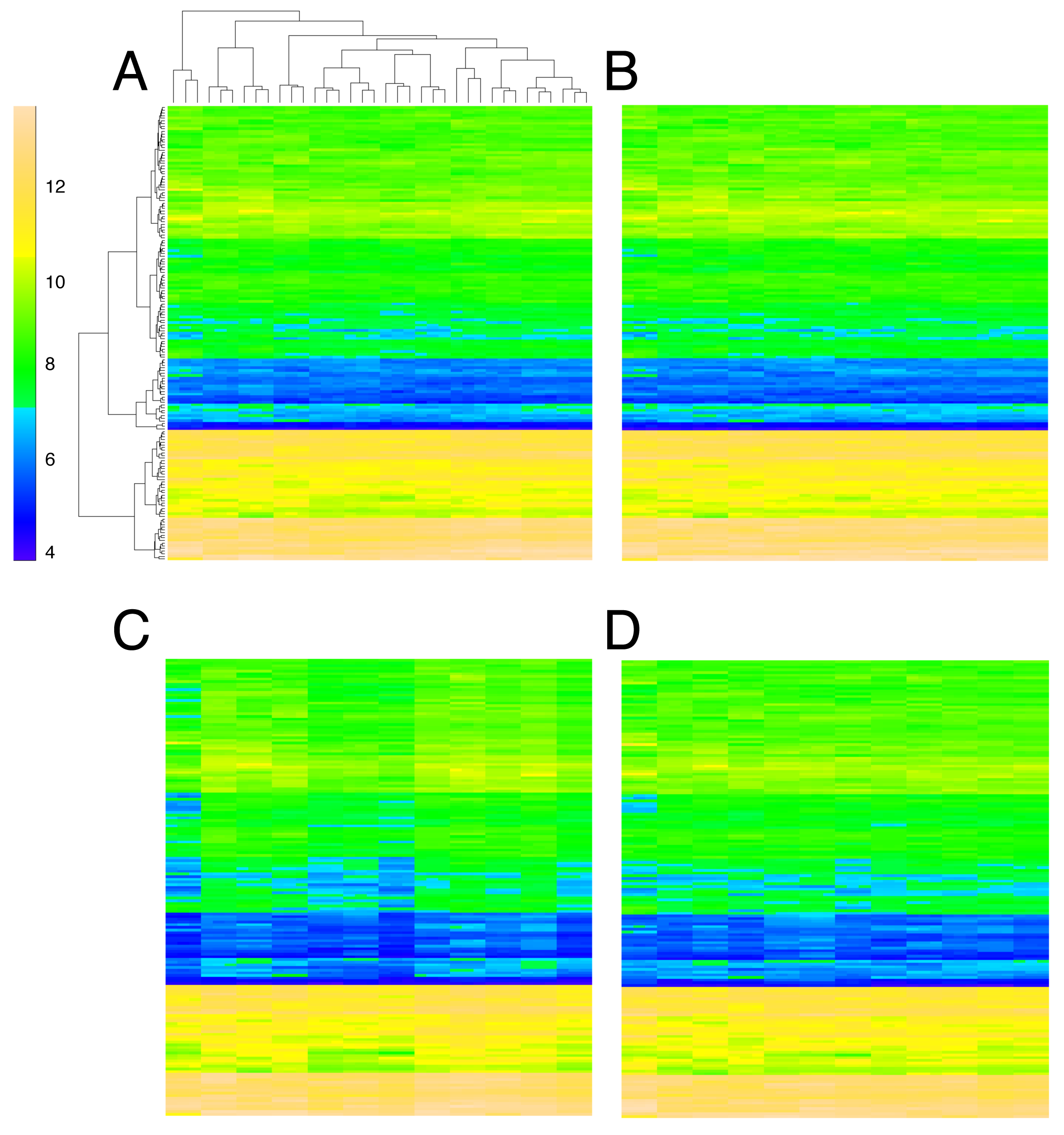

Supplement: Figure S9 — Heatmaps of Arabidopsis invariant gene absolute expression after applying four different normalization algorithms. A, RMA using all 22,810 probe sets on the Affymetrix Arabidopsis ATH1 array. B, SBQ. C, RMAPS. D, RIMS. Normalizations B to D used only a subset of 299 probe sets (37 DEFLs, 171 invariants, 91 marker genes). Expression values have been log2 transformed. Two-dimensional hierarchical agglomerative clustering was applied to the set of 37 DEFLs (rows) and 36 arrays (columns) from the RMA-normalized data in A. This ordering of rows and columns was preserved for panels B to D. All 171 invariant gene probe sets on the ATH1 array are exactly identical to their counterparts on the AtDEFL array. The 36 arrays are the same as those described in Figure S3. (TIF) [file pone.0058992.s009.tif]

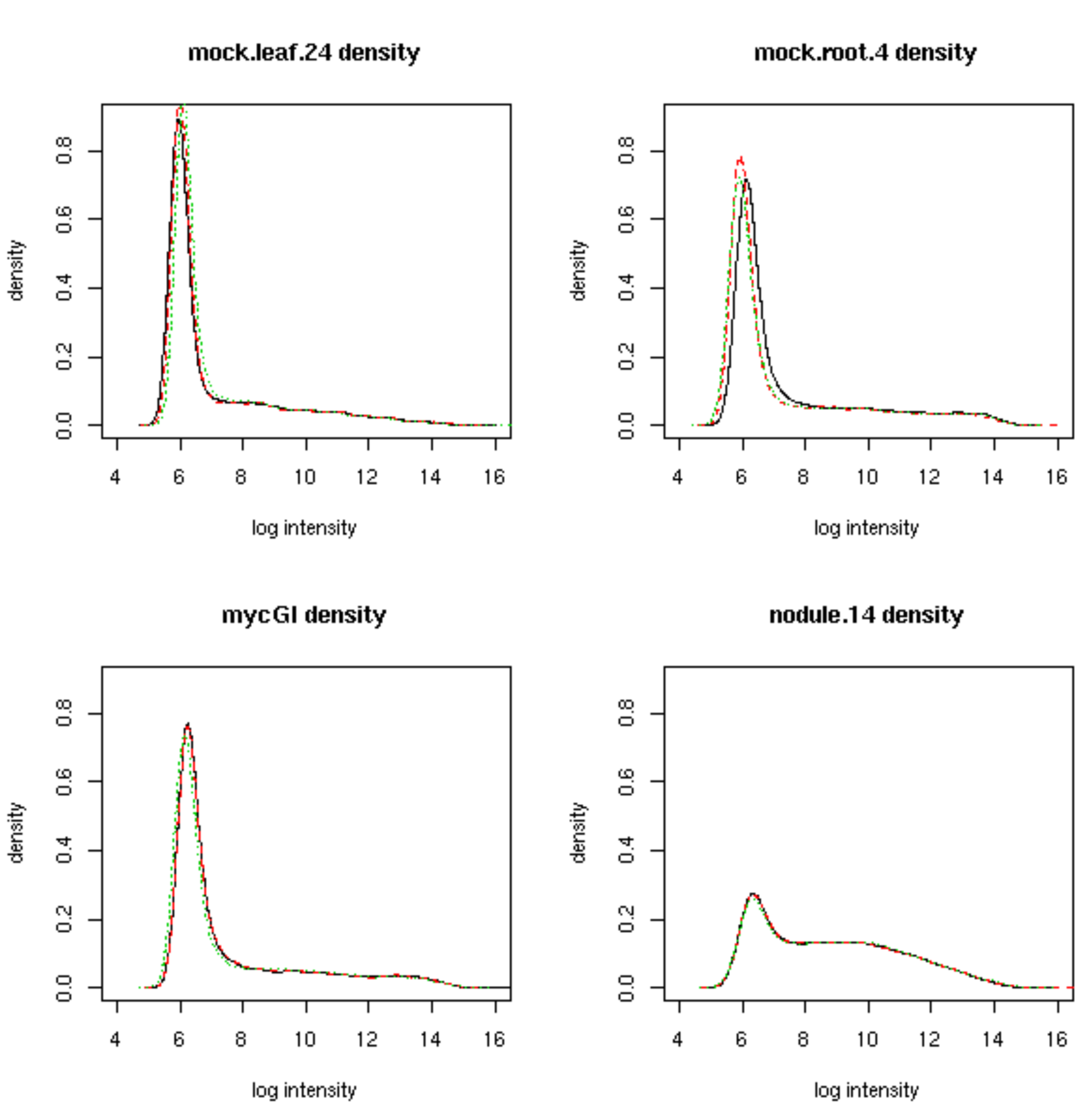

Supplement: Figure S10 — Raw log2 signal intensity distribution plots for MtDEFL array experiments profiling leaves, roots, mycorrhizal roots, and root nodules. The three different color lines represent independent biological replicates. The large peak in these plots at low intensity values typically represents absent probe sets in the noise-level. Systematic shifts in these plots will be reduced or eliminated by most normalization methods, but changes in shape of the curve may reflect real biological differences among the samples. (TIF) [file pone.0058992.s010.tif]

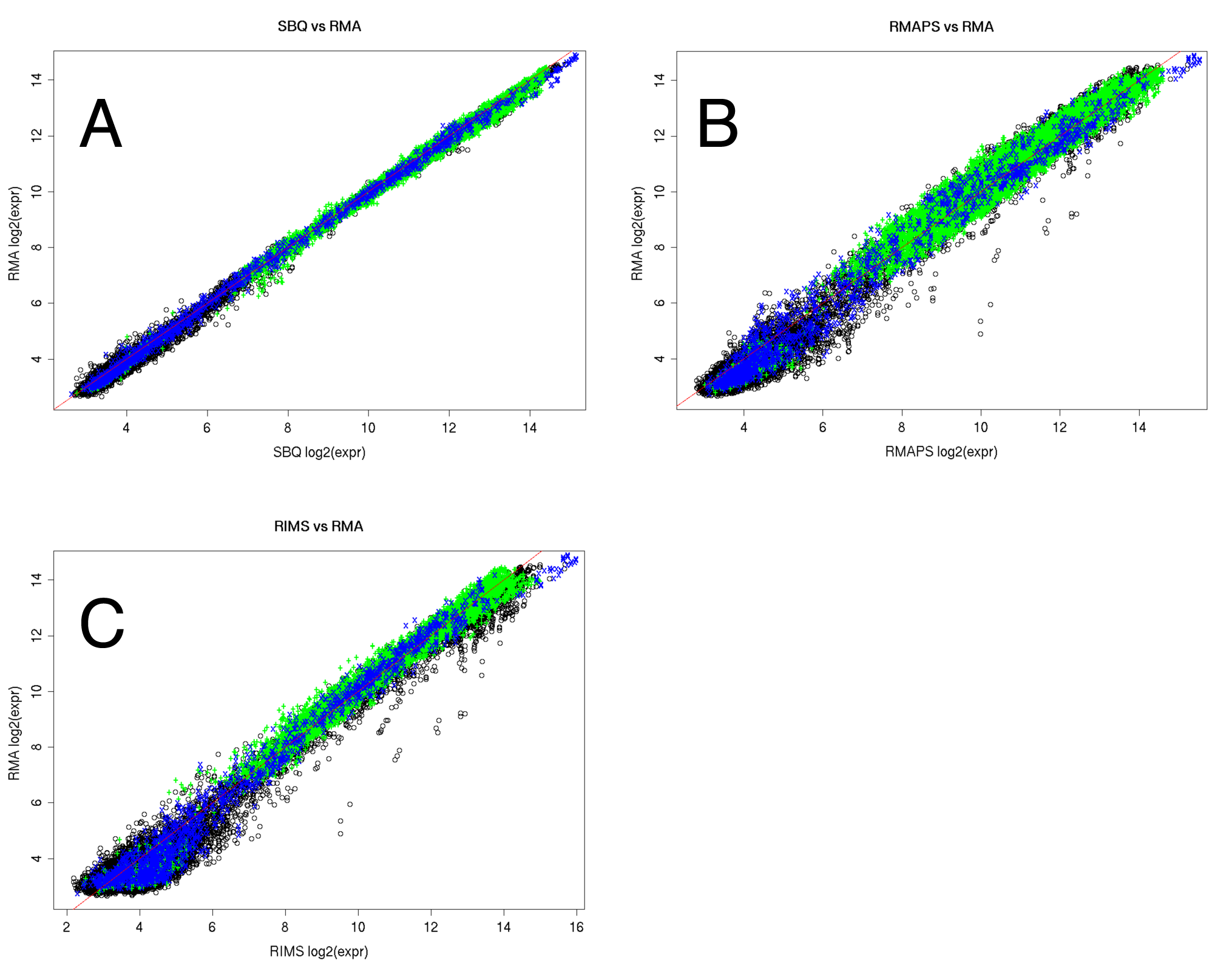

Supplement: Figure S11 — Scatter plots showing correlation of normalized expression values obtained from three boutique array methods as compared to whole-array RMA across 62 MtDEFL arrays. A, SBQ vs. RMA. B, RMAPS vs. RMA. C, RIMS vs. RMA. All three boutique array normalizations used only a subset of 565 probe sets that correspond to genes represented on the MtDEFL array (370 DEFLs - black circles, 172 invariants - green plus signs, 23 marker genes - blue crosses). Each was compared to the reference RMA normalization, which included all 61,278 probe sets on the Affymetrix Medicago array in the normalization process. Expression values have been log2 transformed. All 370 probe sets on the Medicago array that matched a Medicago DEFL with at least six of 11 probe sets were included in the analysis. The 62 arrays included three biological replicates for a wide variety of tissues and conditions obtained from ArrayExpress and GEO: E-MEXP-1097 (flowers, leaves, nodules at various developmental stages, petioles, pods, roots, seeds at various developmental stages, stems and vegetative buds), E-MEXP-1092 (methyl-jasmonate and yeast-elicited cell suspensions and controls at multiple time points), GSE8131 (NAA and BAP-treated leaf explants), and GSE8115 (roots). (TIF) [file pone.0058992.s011.tif]
