# Supplementary material for: Spatio-Temporal Expression Patterns of Arabidopsis thaliana and Medicago truncatula Defensin-Like Genes
Source: PLoS One. 2013 Mar 18;8(3):e58992. doi: 10.1371/journal.pone.0058992 (PMC3601123; doi:10.1371/journal.pone.0058992)
Supplement: Text S1 — Development of the custom AtMtDEFL array and robust data normalization methods. Mapping Medicago probe sets to the latest annotation of the Medicago gene annotation. (DOCX) [file pone.0058992.s024.docx]

**Development of the Custom AtMtDEFL Array and Robust Data Normalization Methods**

The AtMtDEFL array includes probe sets for 317 Arabidopsis *DEFL*s, 15 Arabidopsis *DEFL*-related Genes (*MEG*s) [1], [2], and 684 Medicago *DEFL*s [2], plus additional marker genes. The array also contains probe sets with invariant levels of expression (hereafter called invariant genes) to aid microarray data normalization. Probe sets were interspersed on the custom array, although chip hybridization and microarray data analysis were performed for only one plant species at a time. The subset of the AtMtDEFL array made up of Arabidopsis probe sets is hereafter referred to as the AtDEFL array whereas the subset of the AtMtDEFL array made up of Medicago probe sets is hereafter termed the MtDEFL array.

Although Robust Multi-array Average (RMA) [3]) has been frequently applied to normalize data from genome-wide Affymetrix arrays, the primary assumption that the distribution of probes responding in each quantile level of expression is similar across experiments has not been met by the signal intensity data for the *DEFL* family of genes on the AtDEFL custom array (Figure S5). Distribution plots of the raw signal intensity data were clearly skewed to much higher signal intensity levels in experiments with Arabidopsis siliques and inflorescences than with leaves and roots. If we attempted to apply RMA globally across the AtDEFL custom array, these highly-variable distribution plots would be coerced into an average distribution plot, which would significantly skew absolute expression values and simultaneously normalize out real biological differential gene expression between arrays. These caveats in the distribution of gene expression values are often not available for genome-wide Affymetrix arrays such as ATH1 because the sheer number, genome-wide scale, and random nature of probe sets on these arrays tend not to show marked tissue- and condition-specific effects. Hence, the assumptions underlying RMA are not violated for such larger arrays as ATH1.

The ATH1 and the custom AtDEFL array share probe sets for 299 genes (171 invariants, 37 *DEFL*s, and 91 markers and other genes). As a test for comparing microarray data normalization approaches as described in the Materials and Methods, we downloaded publicly available microarray data and carried out RMA normalization on 36 ATH1 datasets (Figure S6). The public microarray data sets include biological experiments in which various common *DEFL*s were known to be differentially expressed (e.g., flowers, siliques, roots, leaves, seeds, stamens, stems, cotyledons, and seedlings). Restricting our view to the subset of the 299 genes on the ATH1 array that were shared by the custom AtDEFL array, we sought a normalization method that, when applied only to these probe sets, achieved high correlation with the expression values obtained via RMA performed on the entire ATH1 array.

The Stable-Based Quantile (SBQ; [4]) method proved to be an outstanding correlation coefficient (R^2^ = 0.998) with RMA, even though it only includes expression data from the restricted set of *DEFL*s, invariants, and other genes common to the AtDEFL custom array (Figure 1). Two other microarray data normalization methods proposed, RMA with Invariant Median Scaling (RIMS) and RMA with Median Absent Probe set Scaling (RMAPS) (see Materials and Methods for details), also showed high correlation coefficients of R^2^ = 0.992 and R^2^ = 0.998, respectively with RMA. Heatmaps of AtDEFLs, invariants, and additional genes produced by all four normalization methods examined are presented in Figures S7 to S9.

Similarly, the MtDEFL custom array raw probe set intensity distributions in experiments with root nodules were highly skewed to higher intensities relative to uninoculated roots and other Medicago tissues and conditions examined (Figure S10). We concluded that the assumption underlying quantile normalization across MtDEFLs using the custom array does not apply. The Affymetrix Medicago genome array and the custom MtDEFL array share probe sets for 565 Medicago genes (172 invariants, 370 *DEFL*s, and 23 additional genes). When applying the SBQ, RMAPS and RIMS normalizations to the standard Affymetrix Medicago array, restricting the analysis to the 565 genes that are shared with the custom MtDEFL array, we again achieve outstanding correlation with the expression values obtained from RMA applied on more than 61,000 probe sets (Figure S11). SBQ also showed the best concordance with RMA among the three normalization methods examined, although all three normalization methods showed high correlation coefficients (R^2^ = 0.99) with RMA.

We performed SBQ, RIMS, and RMAPS normalization of the AtDEFL array data for untreated roots, leaves, and inflorescences and computed the correlation with data deposited at NCBI for similar Arabidopsis samples across the subset of shared genes on the two microarray platforms. All raw public data from NCBI was re-normalized using RMA prior to comparison. We found that the AtDEFL custom array performs similarly to the standard Affymetrix ATH1 array. SBQ correlation coefficients with ATH1 data sets were outstanding ranging from a low of R^2^ = 0.93 for inflorescences to R^2^ = 0.95 for roots (Figure 2). Correlation coefficients between RIMS and RMAPS AtDEFL data sets and the ATH1 RMA datasets were nearly as good, each with R^2^ = 0.93 or R^2^ = 0.94 across data sets from these plant organs. Consequently, we used SBQ to normalize AtMtDEFL custom array datasets in the subsequent study.

**Mapping Medicago Probe sets to the Latest Annotation of the Medicago Gene Annotation**

At the time the AtMtDEFL array was commissioned at Affymetrix, the Arabidopsis genome was complete and all 317 DEFLs were properly annotated at TAIR. Thus the ‘_x_at’ designation faithfully indicated whether a probe set had one or more probes that matched a gene that was not the primary target (i.e., cross-hybridizing). Also, the ‘_s_at’ accurately indicated that multiple family members (nearly always two or three paralogs) were exactly matched by all probes in the probe set.

At that time, however, there was no primary assembly of the Medicago genome. Instead, we had to construct probe sets from a compilation of our gene predictions in the scattered (sometimes redundant) BAC sequences and the assembled transcript contigs that were available. This is non-ideal, but unavoidable at the time, because excess redundancy (contigs or BAC sequences that correspond to the same genome locus) could not have been removed. Indeed, of the 684 MtDEFL probe sets there are 261 (38%) annotated as possibly cross-hybridizing (i.e., with ‘_x_at’ suffix) and 102 (15%) as possibly reporting the expression of multiple genes (i.e., with ‘_s_at’ suffix). We show below that both numbers are gross over-estimates.

Now that much of the genome is assembled and the DEFL genes in the current draft (Mt3.5) have been identified [5], we can map the actual probe sequences to the current IMGAG gene transcript sequences (Mt3.5v5 http://jcvi.org/cgi-bin/medicago/download.cgi). We have done this using bowtie [6]. Probes from 537 of the 684 probe sets (79%) map to the IMGAG genes. This a little lower than the percentage of a typical expressed gene [5], but is in line with expectations since only the gene-rich portion of the genome has been sequenced [5] and many DEFLs have been shown to be near repeats [7], [8]. Of the 537 probe sets that map to genes, 467 (87%) have at least six probes matching the target gene. We expect this to be less than the 11 designed probes in many cases because the annotated IMGAG gene boundaries are not complete and lack portions of the 3’-UTR that is prioritized in Affymetrix designs. Most importantly, 440 of the 537 probe sets (82%) exactly match a single IMGAG gene. Roughly the same percentage (379/467=81%) uniquely match a single gene if we restrict our view to those probe sets that have at least six probes matching the gene. Of those that do not map to a unique gene, 60 out of 537 (11%) cross-hybridize and 37 (7%) have all their probes mapping exactly to multiple genes (two or three paralogs in all cases).

We believe these numbers are impressive for such a highly redundant gene family, and this is due primarily because Affymetrix was able to specifically design the probes to match the desired targets and specifically avoid the selection of cross-hybridizing probes wherever possible. Further, although RNA-seq is revolutionizing gene expression profiling, cross-mapping of reads is likely to be a more significant problem with that technology than with our custom-designed array. This is because read placement in RNA-seq is random, and close gene family members will result in ambiguous mapping and poor phred-scale mapping qualities (MAPQ=0), a significant problem with gene quatification in RNA-seq [9], [10].

**Discussion and Conclusion**

Microarray data normalization algorithms such as Affymetrix's MAS 5.0 [11] or loess [12] require that the majority of genes show an unchanged pattern of gene expression among the conditions under consideration, or at least that an equivalent proportion of genes are up- and down-regulated. Alternatively, RMA and other quantile normalization based schemes [3], [13] require that the density distribution of intensities is at least qualitatively similar across samples. These essential probe intensity distribution assumptions for microarray data are clearly violated when analyzing data from boutique arrays such as the custom AtMtDEFL chip used in this study. For example, we observed more than 500 Medicago *DEFL*s expressed in nitrogen-fixing root nodules, yet only a minority was detectable in uninoculated roots. These extreme expression patterns pose a challenge to routine microarray data normalization algorithms [4], [14]. Wilson et al. [14] proposed that design of boutique arrays should include a set of invariant housekeeping genes for accurate data normalization purposes. Hence the AtMtDEFL custom array design included more than 170 invariant genes for each plant species to aid in the microarray data normalization steps, plus numerous condition-specific marker genes to serve as positive controls. Ideally, invariant genes should be chosen such that the full range of observed expression levels for the genes of interest is uniformly covered. Wilson et al. [14] removed intensity-dependent nonlinear trends in the data by applying loess normalization to the invariant genes and then transforming the variable genes of interest by interpolating from the flanking invariant genes. Genes whose expression fell outside the invariant genes range could be normalized via linear extrapolations off the lower and upper edges in the transformation curves. Sato et al. [4] also used a similar approach, the Stable-gene's Based Quantile (SBQ) normalization, which uses a quantile normalization scheme in place of the loess normalization used [14]. In this study, we found that the SBQ method performs superbly, closely mirroring the expression values that RMA produces, when the latter is used with sufficient additional genes to avoid violation of its central assumption of similar intensity distribution across conditions [3].

Two additional normalizations were examined: RIMS and RMAPS. Both of these methods perform standard RMA separately within biological replicates, and then scale the values obtained between these independently normalized sets differently. RIMS scales each biological replicate set using the median expression value of the set of approximately 170 invariant genes. RMAPS uses the median expression value of the set of genes that are “Absent” (MAS 5.0; [11]) across all conditions in the study. Also, both RIMS and RMAPS have the significant drawback that they artificially reduce the within biological-group variance, and correspondingly accentuate the between-group variance. Hence, if accurate differential expression between groups is sought, use of these methods should be discouraged. Therefore, SBQ proved to be the best microarray data normalization methods for the custom AtMtDEFL array.

**Literature Cited**

1. Gutierrez-Marcos JF, Costa LM, Biderre-Petit C, Khbaya B, O’Sullivan DM, et al. (2004) *Maternally expressed gene1* is a novel endosperm transfer cell-specific gene with a maternal parent-of-origin pattern of expression. Plant Cell 16: 1288-1301.
2. Silverstein KAT, Moskal WA, Wu HC, Underwood BA, Graham MA, et al. (2007) Small cysteine-rich peptides resembling antimicrobial peptides have been under-predicted in plants. Plant J 51: 262-280
3. Irizarry RA, Hobbs B, Collin F, **Beazer-Barclay YD, Antonellis KJ, et al.** **(2003)** Exploration, normalization, and summaries of high density oligonucleotide array probe level data. Biostatistics 4: 249-264.
4. Sato M, Mitra RM, Coller J, Wang D, Spivey NW, et al. (2007) A high-performance, small-scale microarray for expression profiling of many samples in Arabidopsis-pathogen studies. Plant J 49: 565-577.
5. Young N, Debellé F, Oldroyd G, Geurts R, Cannon S, et al. (2011) The Medicago genome provides insight into the evolution of rhizobial symbioses. Nature 480: 520-524.
6. Langmead B, Trapnell C, Pop M, Salzberg SL (2009) Ultrafast and memory-efficient alignment of short DNA sequences to the human genome. Genome Biol 10: R25.
7. Graham MA, Silverstein KAT, Cannon SB, VandenBosch KA (2004) Computational identification and characterization of novel genes from legumes. Plant Physiol 135: 1179-1197.
8. Silverstein KAT, Graham MA, Paape TD, VandenBosch KA (2005) Genome organization of more than 300 defensin-like genes in Arabidopsis. Plant Physiol 138: 600-610.
9. [Li B](http://www.ncbi.nlm.nih.gov/pubmed?term=Li%20B%5BAuthor%5D&cauthor=true&cauthor_uid=20022975), [Ruotti V](http://www.ncbi.nlm.nih.gov/pubmed?term=Ruotti%20V%5BAuthor%5D&cauthor=true&cauthor_uid=20022975), [Stewart RM](http://www.ncbi.nlm.nih.gov/pubmed?term=Stewart%20RM%5BAuthor%5D&cauthor=true&cauthor_uid=20022975), [Thomson JA](http://www.ncbi.nlm.nih.gov/pubmed?term=Thomson%20JA%5BAuthor%5D&cauthor=true&cauthor_uid=20022975), [Dewey CN](http://www.ncbi.nlm.nih.gov/pubmed?term=Dewey%20CN%5BAuthor%5D&cauthor=true&cauthor_uid=20022975) (2009) RNA-Seq gene expression estimation with read mapping uncertainty. Bioinformatics 26: 493-500.
10. [Trapnell C](http://www.ncbi.nlm.nih.gov/pubmed?term=Trapnell%20C%5BAuthor%5D&cauthor=true&cauthor_uid=20436464), [Williams BA](http://www.ncbi.nlm.nih.gov/pubmed?term=Williams%20BA%5BAuthor%5D&cauthor=true&cauthor_uid=20436464), [Pertea G](http://www.ncbi.nlm.nih.gov/pubmed?term=Pertea%20G%5BAuthor%5D&cauthor=true&cauthor_uid=20436464), [Mortazavi A](http://www.ncbi.nlm.nih.gov/pubmed?term=Mortazavi%20A%5BAuthor%5D&cauthor=true&cauthor_uid=20436464), [Kwan G](http://www.ncbi.nlm.nih.gov/pubmed?term=Kwan%20G%5BAuthor%5D&cauthor=true&cauthor_uid=20436464), et al. (2010) Transcript assembly and quantification by RNA-Seq reveals unannotated transcripts and isoform switching during cell differentiation. Nat Biotechnol 8: 511-515.
11. Liu WM, Mei R, Di X, Ryder TB, Hubbell E, et. al (2002) Analysis of high density expression microarrays with signed-rank call algorithm. Bioinformatics 18: 1593-1599
12. Smyth GK, Speed T (2003) Normalization of cDNA microarray data. Methods 31: 265-273.
13. **Bolstad BM, Irizarr RA, Åstrand M, Speed TP (2003)** A comparison of normalization methods for high density oligonucleotide array data based on variance **and** bias. Bioinformatics 19: 185-193.
14. Wilson DL, Buckley MJ, Helliwell CA, Wilson IW (2003) New normalization methods for cDNA microarray data. Bioinformatics 19: 1325-1332.
